# Supplementary material for: Concentration of heavy metals in pasteurized and sterilized milk and health risk assessment across the globe: A systematic review
Source: PLoS One. 2024 Feb 5;19(2):e0296649. doi: 10.1371/journal.pone.0296649 (PMC10843077; doi:10.1371/journal.pone.0296649)
Supplement: S1 Table — (DOC) [file pone.0296649.s002.doc]

Table S2. Quality assessments of the included studies based on Newcastle–Ottawa scale

| **Code** | **Authors/years** | **Standard sampling protocol** | **Period of sampling** | **QA/QC conducted** | **All objectives achieved** | **Report on sources** | **Mean metals levels** | **Overall quality score** |
| --- | --- | --- | --- | --- | --- | --- | --- | --- |
| 1 | ZH Shar et al.(2021) | No | Yes | Yes | Yes | Yes | Yes | 5 |
| 2 | AtigaElbagermi et al.(2020) | Yes | Yes | Yes | Yes | No | Yes | 5 |
| 3 | Feizi,R et al.(2020) | No | Yes | Yes | Yes | No | Yes | 4 |
| 4 | Noaman,V et al.(2020) | Yes | Yes | Yes | Yes | Yes | Yes | 6 |
| 5 | Sharifi,Sh et al. (2020) | No | Yes | Yes | Yes | Yes | Yes | 5 |
| 6 | Almayahi B et al.(2019) | No | Yes | Yes | Yes | No | Yes | 4 |
| 7 | Eman N. Abdelfatah(2019) | No | Yes | Yes | Yes | No | Yes | 4 |
| 8 | Fakhreddini ,M et al.(2019) | No | Yes | Yes | Yes | No | Yes | 4 |
| 9 | Mostafa, M et al.(2018) | No | Yes | Yes | Yes | No | Yes | 4 |
| 10 | E Harlia (2017) | Yes | Yes | Yes | Yes | No | Yes | 5 |
| 11 | Fatma Esra Totan (2017) | Yes | Yes | Yes | Yes | Yes | Yes | 6 |
| 12 | Ahmed,M et al.(2016) | No | Yes | No | Yes | No | Yes | 3 |
| 13 | Beikzadeh (2016) | No | Yes | Yes | Yes | No | Yes | 4 |
| 14 | Naema A. Abdeljalil (2016) | Yes | Yes | Yes | Yes | No | Yes | 5 |
| 15 | Sadeghi,N et al.(2016) | No | Yes | Yes | Yes | No | Yes | 4 |
| 16 | Shahbazi,Y et al.(2016) | No | Yes | Yes | Yes | No | Yes | 4 |
| 17 | Akhtar.S(2015) | No | Yes | Yes | Yes | No | Yes | 4 |
| 18 | MadaniTonekaboni (2015) | No | Yes | Yes | Yes | No | Yes | 4 |
| 19 | Marcio Augusto et al.(2015) | Yes | Yes | Yes | Yes | No | Yes | 5 |
| 20 | Meijuan Yu et al.(2015) | Yes | Yes | No | Yes | No | Yes | 4 |
| 21 | Sahar Y Issa et al. (2015) | No | Yes | Yes | Yes | No | Yes | 4 |
| 22 | Santos, C. B. G et al. (2014) | No | Yes | Yes | Yes | No | Yes | 4 |
| 23 | Sobhanardakani (2014) | No | Yes | No | Yes | No | Yes | 3 |
| 24 | Suturovic´.Z et al. (2014) | No | Yes | Yes | Yes | No | Yes | 4 |
| 25 | Ghezelbash,M et al. (2013) | Yes | Yes | No | Yes | No | Yes | 4 |
| 26 | Jianfeng Ping(2013) | No | Yes | Yes | Yes | No | Yes | 4 |
| 27 | NaserAlibeigi (2013) | No | Yes | Yes | Yes | No | Yes | 4 |
| 28 | Rezaei,M et al. (2013) | Yes | Yes | Yes | Yes | Yes | Yes | 6 |
| 29 | Shahbazi,Y et al. (2013) | No | Yes | Yes | Yes | No | Yes | 4 |
| 30 | Lukáčová et al. (2012) | No | Yes | Yes | Yes | No | Yes | 4 |
| 31 | Naeem Khan (2012) | No | Yes | Yes | Yes | No | Yes | 4 |
| 32 | Salah Fathy Aal et al. (2012) | No | Yes | Yes | Yes | No | Yes | 4 |
| 33 | Miclean,M et al.(2011) | Yes | Yes | Yes | Yes | No | Yes | 5 |
| 34 | Clarissa S. P (2010) | No | Yes | Yes | Yes | No | Yes | 4 |
| 35 | Lutfullah.G (2009) | No | Yes | Yes | Yes | No | Yes | 4 |
| 36 | Birghila,S et al.(2008) | No | Yes | No | Yes | No | Yes | 3 |
| 37 | Abou Arab, A.A.K et al.(2007) | No | Yes | Yes | Yes | No | Yes | 4 |
| 38 | Pohl.P(2007) | No | Yes | Yes | Yes | No | Yes | 4 |
| 39 | Gul Kazi,T et al. (2006) | No | Yes | Yes | Yes | No | Yes | 4 |
| 40 | li-qiang qin et al.(2006) | Yes | Yes | Yes | Yes | No | Yes | 5 |
| 41 | Perveen.F (2005) | No | Yes | No | Yes | No | Yes | 3 |
| 42 | Soares,A et al. (2004) | No | Yes | No | Yes | No | Yes | 3 |
| 43 | TOKUSüOGÿ LU,O et al. (2002) | No | Yes | Yes | Yes | No | Yes | 4 |
| 44 | Martino,F (2001) | No | Yes | No | Yes | No | Yes | 3 |
| 45 | ELENA M et al. (1999) | No | Yes | No | Yes | No | Yes | 3 |
| 46 | Tripathi.(1999) | No | Yes | Yes | Yes | No | Yes | 4 |
| 47 | E. M. RodrmHguez (1995) | No | Yes | No | Yes | No | Yes | 3 |
| 48 | G. Zurera-Cosano (1993) | No | Yes | Yes | Yes | No | Yes | 4 |
