# Supplementary material for: Concentration of heavy metals in pasteurized and sterilized milk and health risk assessment across the globe: A systematic review
Source: PLoS One. 2024 Feb 5;19(2):e0296649. doi: 10.1371/journal.pone.0296649 (PMC10843077; doi:10.1371/journal.pone.0296649)
Supplement: Attachment — Submitted filename: 17.7.docx [file pone.0296649.s003.docx]

The article is very neat and orderly, and sufficient and accurate sources have been selected.

Only a few points can be corrected.

Do not use **we** Example: Line 153, “we used,” Line 156,” we included”, Line 320, “we retrieved,” Line 154“In this review we” should be omitted.

Tables must be modified. It should have three main lines and numbers.

Symbols should be used instead of color numbers and explained at the end of the table.

Why didn't you do a meta-analysis? It can help the decision-making power to solve this problem and provide better results. The following article is one of the examples of Iran that is recommended to be used, which was not seen in your sources.

A systematic review and meta-analysis of lead and cadmium concentrations in cow milk in Iran and human health risk assessment. Environmental Science and Pollution Research. 2020 Apr;27:10147-59.
