# Supplementary material for: Concentration of heavy metals in pasteurized and sterilized milk and health risk assessment across the globe: A systematic review
Source: PLoS One. 2024 Feb 5;19(2):e0296649. doi: 10.1371/journal.pone.0296649 (PMC10843077; doi:10.1371/journal.pone.0296649)
Supplement: Attachment — Submitted filename: review article.doc [file pone.0296649.s004.doc]

***Concentration of Heavy Metals in Pasteurized and sterilized Milk and Health Risk Assessment across the Globe: A Systematic Review***

**Zahra Alinezhad^1^, Mohammad Hashemi^1,2^, Seyedeh Belin Tavakoly Sany^3, 4*^**

1. Department of Nutrition, Faculty of Medicine, Mashhad University of Medical Sciences, Mashhad, Iran
2. Medical Toxicology Research Center, Mashhad University of Medical Sciences, Mashhad, Iran
3. Department of Health Promotion and Education, School of Health Mashhad University of Medical Sciences, Mashhad, Iran
4. Social Determinants of Health Research Center, Mashhad University of Medical Sciences, Mashhad 13131-99137, Iran

-----------------------------------------------------------------------------------------------------

*****Corresponding author: Seyedeh Belin Tavakoly Sany (Email: [belintavakoli332@gmail.com](mailto:belintavakoli332@gmail.com) ,tavakkolisanib@mums.ac.ir ,Tell:989150060122)

# Abstract

Objective: Although milk and dairy products are almost complete food, they can contain toxic heavy elements with potential hazards for consumers.  This review aims to provide a comprehensive report on the occurrence, concentration, and health risk of selected heavy metals in the pasteurized and sterilized milk recorded across the world.

Methods: The Preferred Reporting Items for Systematic Reviews and Meta-Analysis (PRISMA) was used to develop this systematic review. Databases included the Web of Knowledge, Scopus, Scientific Information Database ~~(SID)~~, Google scholar and PubMed from inception until January 2023. Keywords related to the term “Heavy metals”, “Arsenic” and “Pasteurized and sterilized milk” and “Risk Assessment” were used. The potential health risks to human health from milk daily consumption were estimated using extracted data on heavy metals concentration based on Metal Estimated Daily Intake (EDI), Target hazard quotient (THQ), and carcinogenic risk.

Results: A total of 48 potentially relevant articles with data on 981 milk samples were included in the systematic review. Atomic Absorption Spectroscopy (AAS), ICP-MS, and ICP-OES was the most common valid method used to measure heavy metals in milk in the globe. Cupper, Cd, Zn, and Pb were the most common contaminants, which exceeded the maximum permissible criteria in 94%, 67%, 62%, and 46% of the milk samples tested. Estimated values for THQ and CR showed that milk consumers may exposed to moderate to high level of health risk through the continuous use of pasteurized and sterilized milk.

Conclusion: The elevated levels of all heavy metals in milk samples, calls for maximum control and strict regulation by environmental and health protection. Further studies are required to develop safe milk processing and handling methods for the decontamination of heavy metals in milk and its products.

Keyword: *heavy metals, milk, carcinogenic risk, food safety, Public health*

# Introduction

Food security is an important challenge in the maintenance of people's health worldwide for disease control and prevention and protection against environmental pollution(1, 2). As defined by the World Health Organization (WHO) and Food and Agriculture Organization (FAO), food security is the control and observation of criteria during food production, preservation, and distribution processes to ensure that healthy and flawless food is consumed by humans(2).

Milk and dairy products are the main sources of macro- and micronutrients, such as essential fatty acids, amino acids and vitamins, which are nesessary for bone development, growth, and immune functions(3-5). Several studies have reported that consuming at least three of dairy products per day has a beneficial impact on energy and nutrient intakes as well as of vitamin D, magnesium, and calcium, compared with intakes of people who consumed fewer servings of dairy products per day(6). In last decade, milk consumption has increased by about 20% globally, and 48% of the total milk products were represented by Bovine milk. Milk and dairy products can contain residues of hazardous chemical or biological pollutants with potential hazards for consumers(7-9). The risk of biological pollutants derives from cattle milking includs the exposure of udders to the environmental pollutants, storage, equipment, and dirty pipes. Chemical pollution of milk comes from use of illegal or legal veterinary products, forages and feed contaminated with natural toxin, application of agrochemicals, and the improper use of chemicals during processing, production, packaging, storage, and handeling, storage, and even pasteurization of milk(10, 11). Based on the literature, the most reported contaminantes that treatens the safety of milk are pathogenic micoorganisms (14.57%), heavy metals (22.18%), antibiotics (22.18%), pesticides(22.05%), and mycotoxins(9.97%). Therefore, heavy metals are stand out among contaminants.

Stable chemical pollutants such as heavy metals are the major food contaminants in the world. Heavy metals are elements with a high specific gravity or atomic number and are attributed to have a specific gravity of > 5-6 g/cm^3^ or an atomic number of 63.5-200 g/mol(7, 12, 13). Some heavy metals like Fe, Cu, Zn, and Mn, are essential elements for human health with critical role in metabolism and biochemical functions in the human body; but their consumption at concentrations higher than the sanitary recommendations imposes adverse effects on human health in terms of toxicology(13-15). Other heavy metals, including Pb, Cd, Hg, and As, are non-essential and toxic elements and can cause adverse effect on human health even at low level(8, 16-18). Heavy metals have been of interest due to such properties as toxicity, high dispersion, high thermal resistance, high level of bioaccumulation along the food chain, and their non-biodegradability(13, 14, 16, 19). At high concentrations, these stable pollutants can have adverse effects, such as toxicity, mutagenesis, and carcinogenesis(8, 16-18). The International Agency for Research on Cancer (IARC) has classified Cd, As, and Pb as carcinogens in groups 1 and 2, respectively(3). The use of heavy metal-contaminated food is a major entry route of these contaminants into the body(3).

Although milk and dairy products are almost complete food, they can contain toxic heavy elements with potential hazards for consumers. It was evidenced that the presence of heavy metals in milk and dairy products was detected in different countries across the world. However, the level of heavy metals concentration is not differed and is constant depending on animal’s nutrition, environmental condition, exposure routes, animal breed, and stage of lactation(3, 12). Several sources contaminate milk and dairy products with heavy metals that mainly originate from environmental pollution that contaminate water and animal feeding, anthropogenic activity, during the packaging and storage of milk products(7-9). Moreover, raw milk is prone to contamination with metals in the factory during different milking processes, including equipment contamination. These metals are somewhat reduced by such procedures as fat separation during pasteurization and sterilization processes, but they are not removed completely(8, 11). Although, pasteurization has remained as an efficient treatment method to eliminate contaminants, many infectious diseases, associated with pasteurized milk occur in raw milk with an exaggerated contamination of chemical and biological compound(20, 21). The main problem in pasteurization is that this method only efficient for reducing the levle of most biological and non-chemical contaminants(22). Based on the literature, very few alternative methods in the of chemical contaminants in cow's milk, cause to relevent analysis to ensure their sufficient quality in the milk products(20). In recent decades several studies reported the occurrence of heavy metals contamination in milk even in pasteurized milk products(7-9). However,  most of these studies have not provided comprehensive reports and trends on the distribution of heavy metals contamination and their health risk in pasteurized and sterilized milk across the world. Public health need to be well-informed about levels of heavy metal contamination and possible cancer or non-cancer risks presented in milk and dairy products. Therefore, this systematic review aims to provide a comprehensive report on the occurrence and concentration, of selected heavy metals including aluminum (Al), copper (Cu), iron (Fe),  Zinc (Zn), Cobalt (Co), and nickel (Ni) as well as toxic heavy metals involving Arsenic (As), cadmium (Cd), mercury (Hg), lead (Pb), and Chromium (Cr) in the pasteurized and sterilized milk recorded across the world. In addition, the potential carcinogenic and non-carcinogenic risks to human health from milk daily consumption were estimated using extracted data on heavy metals concentration.

# 2. Materials and methods

## 2.1. Study Design

We used the Preferred Reporting Items for Systematic Reviews and Meta-Analysis (PRISMA) to develop this systematic review(23). The main outcome of interest for the review the concentrations of heavy metals and trace elements in the pasteurized and sterilized milk reported across the world and potential health risk to human health (carcinogenic and non-carcinogenic effect) and trace elements estimated daily intake of milk were also extracted from different studies. In this review we

## 2.2. Search Strategy

In this study, databases were searched, including the Web of Knowledge, Scopus, Scientific Information Database (SID), Google scholar and PubMed from inception until January 2023. The search was independently conducted for each database, and references of studies were cross-checked for identification of literature that related to concentration of heavy metals in Pasteurized and sterilized milk and health risk assessment across the globe. The reference list of all eligible studies was also hand-searched to find other relevant studies that may have been ignored by the search process. We used keywords related to the term “heavy metals”, “Arsenic” and “Pasteurized and sterilized milk” and “Risk Assessment”.

## 2.3. Inclusion and Exclusion Criteria

We included studies in this review that carried out in world, published in the Persian and English language, reported concentration of heavy metals in Pasteurized and sterilized milk and health risk assessment across the globe, and focused on subjects such as the concentration of heavy metals and health risk assessment of heavy metals in world. The study selection for inclusion eligibility was conducted by scanning the titles, abstracts, and full texts of retrieved articles. All review studies and duplicate researches were excluded.

## 2.4. Data extraction and quality assessment

In this study, a set of basic data were extracted as follow: the publication year/author, study objective, study area, period of sampling, sample size type(s) of samples, quality control/quality assurance, analytical technique, number of heavy metals and heavy metals concentration, and potential pollution sources. The quality of the selected studies was examined via Ofori and Cobbina adapted the Newcastle–Ottawa Scale (NOS). This scale includes six items that were provided with either a “no” or “yes” response to make a general score for each study (Table 2).

## Risk assessment analysis

In this review health risk assessment was conducted based on Metal Estimated Daily Intake (EDI), Target hazard quotient (THQ), and carcinogenic risk, which was calculated based on the mean levels of heavy metals that reviwed in the current review (Table1).

Table1. Health risk assessment indices and variables based on EPA model(24)

| **Risk Indices** | **Formula** | **Variables** | **Cutoff point** |
| --- | --- | --- | --- |
| The value is not exceeded provisional tolerable daily intake (PTDI) of heavy metals. | Concentration (C, mg/kg), Ingestion Rate (IR, gr/kg/day), Exposure frequency (EF, days per year ) equal 365, Exposure duration (ED, years) is 70 and 6 years for adult and child; Body Weight (BW, Kg); Averaging time (AT, day) |  | Estimated Daily Intake (mg/kg/day) |
| Safe: 1>THQ; Possible non-cancer risk and need  Action: 1< THQ | Oral Reference Dose (RFD, mg/kg/day) is equal to As=0.0003, Cd=0.001, Al=0.7, Co=0.0004, Cu=0.04, Fe=0.7, Hg=0.0004, Zn=0.3, Cr=0.003, Ni=0.02, Pb=0.0035, Ba= 0.07 |  | Target Hazard Quotient |
| Safe: 10> HI; Possible non-cancer risk and need  Action: 10< HI |  |  | Hazard Index |
| Safe: 10 -6 >CR; Moderate Risk or Borderline; 10-6 < CR < 10-4; High Risk: 10-4 < CR | Oral Slope Factor (OSF, (mg/kg/day-1) is equal As=1.5, Pb=0.0085, Cd=0.38, Hg=0.0003 |  | Cancer Risk |

# 3. Results and Discussion

## 3.1. Search Outcome and quality assessment

The inclusion and exclusion criteria were implemented to select eligible studies (Figure 1). A total of 1349 published articles were selected for evaluation from which 48 studies with data on 981 samples were met inclusion criteria and included for the final report. All information was extracted based on location, Sample size, apparatus, heavy meals concentration and outcomes. For the quality assessment of eligible articles, about 23% of the studies showed that appropriate quality and ranged from 5 to 6 out of 6(5, 7, 15, 16, 25-31), All these studies used a standard protocol for sampling, period of sampling, quality control, all objectives achieved and mean heavy metals levels. The quality of 29(60.4%) studies was moderate with an average score of 4(3, 8, 12, 14, 17, 18, 32-54), and about 8(16.6%) of the articles showed low quality (from 2 to 3)(4, 19, 55-60) and their major reasons were: a lack of standard protocol for sampling and non-indication of heavy metals sources (Table2).

Table 2. Quality assessments of the included studies based on Newcastle–Ottawa scale

| Code | Authors/years | Standard sampling protocol indicated | Period of sampling | QA/QC conducted | All objectives achieved | Report on sources | Mean metals levels | Overall quality score |
| --- | --- | --- | --- | --- | --- | --- | --- | --- |
| 1 | ZH Shar et al.(2021) | No | Yes | Yes | Yes | Yes | Yes | 5 |
| 2 | AtigaElbagermi et al.(2020) | Yes | Yes | Yes | Yes | No | Yes | 5 |
| 3 | Feizi,R et al.(2020) | No | Yes | Yes | Yes | No | Yes | 4 |
| 4 | Noaman,V et al.(2020) | Yes | Yes | Yes | Yes | Yes | Yes | 6 |
| 5 | Sharifi,Sh et al. (2020) | No | Yes | Yes | Yes | Yes | Yes | 5 |
| 6 | Almayahi B et al.(2019) | No | Yes | Yes | Yes | No | Yes | 4 |
| 7 | Eman N. Abdelfatah(2019) | No | Yes | Yes | Yes | No | Yes | 4 |
| 8 | Fakhreddini ,M et al.(2019) | No | Yes | Yes | Yes | No | Yes | 4 |
| 9 | Mostafa, M et al.(2018) | No | Yes | Yes | Yes | No | Yes | 4 |
| 10 | E Harlia (2017) | Yes | Yes | Yes | Yes | No | Yes | 5 |
| 11 | Fatma Esra Totan (2017) | Yes | Yes | Yes | Yes | Yes | Yes | 6 |
| 12 | Ahmed,M et al.(2016) | No | Yes | No | Yes | No | Yes | 3 |
| 13 | Beikzadeh (2016) | No | Yes | Yes | Yes | No | Yes | 4 |
| 14 | Naema A. Abdeljalil (2016) | Yes | Yes | Yes | Yes | No | Yes | 5 |
| 15 | Sadeghi,N et al.(2016) | No | Yes | Yes | Yes | No | Yes | 4 |
| 16 | Shahbazi,Y et al.(2016) | No | Yes | Yes | Yes | No | Yes | 4 |
| 17 | Akhtar.S(2015) | No | Yes | Yes | Yes | No | Yes | 4 |
| 18 | MadaniTonekaboni (2015) | No | Yes | Yes | Yes | No | Yes | 4 |
| 19 | Marcio Augusto et al.(2015) | Yes | Yes | Yes | Yes | No | Yes | 5 |
| 20 | Meijuan Yu et al.(2015) | Yes | Yes | No | Yes | No | Yes | 4 |
| 21 | Sahar Y Issa et al. (2015) | No | Yes | Yes | Yes | No | Yes | 4 |
| 22 | Santos, C. B. G et al. (2014) | No | Yes | Yes | Yes | No | Yes | 4 |
| 23 | Sobhanardakani (2014) | No | Yes | No | Yes | No | Yes | 3 |
| 24 | Suturovic´.Z et al. (2014) | No | Yes | Yes | Yes | No | Yes | 4 |
| 25 | Ghezelbash,M et al. (2013) | Yes | Yes | No | Yes | No | Yes | 4 |
| 26 | Jianfeng Ping(2013) | No | Yes | Yes | Yes | No | Yes | 4 |
| 27 | NaserAlibeigi (2013) | No | Yes | Yes | Yes | No | Yes | 4 |
| 28 | Rezaei,M et al. (2013) | Yes | Yes | Yes | Yes | Yes | Yes | 6 |
| 29 | Shahbazi,Y et al. (2013) | No | Yes | Yes | Yes | No | Yes | 4 |
| 30 | Lukáčová et al. (2012) | No | Yes | Yes | Yes | No | Yes | 4 |
| 31 | Naeem Khan (2012) | No | Yes | Yes | Yes | No | Yes | 4 |
| 32 | Salah Fathy Aal et al. (2012) | No | Yes | Yes | Yes | No | Yes | 4 |
| 33 | Miclean,M et al.(2011) | Yes | Yes | Yes | Yes | No | Yes | 5 |
| 34 | Clarissa S. P (2010) | No | Yes | Yes | Yes | No | Yes | 4 |
| 35 | Lutfullah.G (2009) | No | Yes | Yes | Yes | No | Yes | 4 |
| 36 | Birghila,S et al.(2008) | No | Yes | No | Yes | No | Yes | 3 |
| 37 | Abou Arab, A.A.K et al.(2007) | No | Yes | Yes | Yes | No | Yes | 4 |
| 38 | Pohl.P(2007) | No | Yes | Yes | Yes | No | Yes | 4 |
| 39 | Gul Kazi,T et al. (2006) | No | Yes | Yes | Yes | No | Yes | 4 |
| 40 | li-qiang qin et al.(2006) | Yes | Yes | Yes | Yes | No | Yes | 5 |
| 41 | Perveen.F (2005) | No | Yes | No | Yes | No | Yes | 3 |
| 42 | Soares,A et al. (2004) | No | Yes | No | Yes | No | Yes | 3 |
| 43 | TOKUSüOGÿ LU,O et al. (2002) | No | Yes | Yes | Yes | No | Yes | 4 |
| 44 | Martino,F (2001) | No | Yes | No | Yes | No | Yes | 3 |
| 45 | ELENA M et al. (1999) | No | Yes | No | Yes | No | Yes | 3 |
| 46 | Tripathi.(1999) | No | Yes | Yes | Yes | No | Yes | 4 |
| 47 | E. M. RodrmHguez (1995) | No | Yes | No | Yes | No | Yes | 3 |
| 48 | G. Zurera-Cosano (1993) | No | Yes | Yes | Yes | No | Yes | 4 |

## 3.2. Study samples characteristics

A total of 48 potentially relevant articles with data on 981 pasteurized and sterilized milk were included in the systematic review. All data were collected from 17 different countries includs, Iran (13:27%)(3, 5, 8, 14, 17, 18, 25, 29, 34, 36, 41, 43, 55) ,Pakistan (6:12.5%)(4, 7, 35, 48, 51, 56), Egypt(5:10%)(12, 33, 38, 46, 49), and Brazil (4:8%)(16, 39, 47, 57) represented the highest number of studies, and the sample size was varied from 5 to 90. Based on results, the first study was conducted in 1993, and 70% of the studies were done in the past five years.

A number of techniques are used to quantified heavy metals in the aqueous phase which related to the desired level of detection and the specific analyte. Our finding showed that from 48 included articles, 15 techniques were used to measure heavy metals in pasteurized and sterilized milk Include; Atomic Absorption Spectroscopy (AAS)(15:31.3%)(7, 12, 26, 33-36, 43, 44, 46, 48-50, 54, 60), Microwave Plasma – Atomic Emission Spectrophotometer(MP-AES)(1:2.1%) (15),Metrohm 797 VA Computrace(1:2.1%) , Inductively Coupled Plasma Atomic Emission Spectroscopy (ICP-AES)(2:4.15%)(19, 25), Graphite Furnace Atomic Absorption Spectroscopy(GFAAS) (2:4.15%)(5, 28), Flame Atomic Absorption Spectrophotometer (FAAS)(3:6.3%)(32, 39, 57), Inductively Coupled Plasma-Mass Spectrometry (ICP-MS)(7:15%)(27, 31, 37, 38, 42, 45, 58), Inductively Coupled Plasma-Optical Emission Spectrometry (ICP-OES)(4:8.5%)(4, 16, 30, 55), polarography apparatus(1:2.1%)(18), voltammeter(1:2.1%)(14), Laser Induced Breakdown Spectroscopy (LIBS)(1:2.1%)(41), Inductively Coupled Plasma Sector Field Mass Spectrometry (ICP-SFMS)(1:2.1%)(29), Anodic Stripping Voltammetry (ASV)(1:2.1%)(47), Electrothermal Atomic Absorption Spectrophotometer(ET-AAS)(2:4.15%)(51, 59) and Metrohm 746 VA trace(1:2.1%)(52), and 5 studies(5:10.5)(3, 17, 40, 53, 56) did not report the Apparatus.

Atomic Absorption Spectroscopy (AAS)(15:31%), ICP-MS, and ICP-OES was the most common valid method used to measure heavy metals in milk in the globe, while other tools were rarely implemented. The atomic absorption spectrophotometer and FAAS are most commonly used to quantify the heavy metals in the aqueous phase such as milk by the developing countries due to its low cost, easy operating process, and rapid detection. But, ICP-MS or ICP-OES have been impelemented for more sensitive measurment of trace elements. However, this method need high costs to run; therefore, it most commonly used in developed countries.

Table 3. Heavy metals levels (Fe, Al, Ni, Cu, Pb, Zn, Hg, Cd, Co, Cr and As) in Pasteurized and sterilized milk reported in research articles published since 1993-2021.

| **Code** | **location** | **N** | **Apparatus** | **Heavy metals levels (mg/L). Mean ± SD** | | | | | | | | | | |
| --- | --- | --- | --- | --- | --- | --- | --- | --- | --- | --- | --- | --- | --- | --- |
|  |  |  |  | **Fe** | **Al** | **Ni** | **Cu** | **Pb** | **Zn** | **Hg** | **Cd** | **Co** | **Cr** | **As** |
| 1 | Pakistan | 10 | AAS | − | − | − | − | 0.006±0.0002 | − | − | 0.002±0.0001 | − | − | − |
| 2 | Libya | 11 | MP-AES | 1.89±16 | − | 0.07±0.01 | 0.1±0.03 | 0.05±0.001 | 2.01±0.19 | − | ND | ND | 0.04±0.001 | − |
| 3 | Iran | 60 | Metrohm 797 VA Computrace | − | − | − | 0.9± 0.6 | 0.02±0.02 | 4.3± 1.6 | − | 0.02± 0.04 | − | − | − |
| 4* | Iran | 25 | ICP-AES | − | − | − | − | 0.008±0.005 | − | − | − | − | − | − |
| 5 | Iran | 18 | GFAAS | − | − | − | − | 0.01±0.003 | − | − | − | − | − | − |
| 6* | Iraqi | - | FAAS | 2.009 | − | − | 0.1 | - | 12.8 | − | 0.4± 0.13 | − | 0.15 | − |
| 7 | Egypt | 20 | AAS | − | − | − | 0.6±0.08 | 1.9±0.2 | − | − | − | − | − | − |
| 8 | Iran | - | − | − | − | − | − | 0.003±0.0006 | − | − | 0.0004±0.0004 | − | − | − |
| 9* | Egypt | 36 | AAS | − | − | 0.07±0.01 | 0.2±0.08 | 0.4±0.1 | 1.4±0.4 | − | 0.003±0.003 | − | 0.07±0.029 | − |
| 10 | Indonesia | 30 | AAS | − | − | − | − | 0.004± 0.002 | − | − | − | − | − | − |
| 11* | Turkey | 40 | ICP-MS | 1.9± 5.7 | 0.06±0.02 | 0.009±0.008 | 0.004±0.002 | − | 0.8±0.3 | − | − | − | 0.01±0.02 | 0.0049±0.002 |
| 12* | Pakistan | 15 | ICP-OES | 1.98 | 3.35 | − | 0.49 | 0.05 | 2.69 | − | 0.004 | − | 0.09 | ND |
| 13* | Iran | 10 | AAS | 3.1±0.005 | − | − | 0.2±0.001 | 0.01±5×10-5 | − | − | 0.0098±5.6×10^-5^ | − | − | 0.007±1.7×10^-5^ |
| 14 | Libya | - | GFAAS | − | − | − | − | 0.01±0.006 | − | − | 0.09 ± 0.1 | − | − | − |
| 15 | Iran | 26 | polarography apparatus | − | − | − | 0.2±0.18 | 0.04±0.03 | 0.9±0.8 | − | 0.049±0.03 | − | − | − |
| 16 | Iran | - | voltammeter | − | − | − | 0.3±0.01 | 0.009±0.0002 | 0.4±0.02 | − | 0.001±0.0001 | − | − | − |
| 17 | Pakistan | 15 | AAS | 1.6±0.05 | − | 0**.14±0.04** | 0.02±0.02 | 0.19±0.1 | 6.8±2.06 | − | 0.04±0.02 | 0.05±0.04 | − | − |
| 18 | Iran | 20 | AAS | − | − | − | − | 0.04±10-6 | − | − | 0.006±10-6 | − | − | − |
| 19 | Brazil | 11 | ICP-OES | 1.3±.4.1 | − | **0.15±0.3** | 0.2±0.15 | 3.5±3.3 | 4.6±3.8 | 6.8±3.2 | − | 1.05±0.8 | − | − |
| 20 | China | 9 | ICP-MS | − | 0.17±0.09 | 0.05±0.08 | 0.04±0.01 | 0.006±0.002 | 2.8±0.4 | − | 0.0019±0.001 | 0.0038±0.0007 | 0.01±0.003 | − |
| 21* | Egypt | 90 | ICP-MS | − | 1.4±0.13 | − | 1.9±0.7 | 3.5±1.5 | 7.7±2.9 | − | 0.29±0.1 | − | − | − |
| 22* | Brazil | 9 | FAAS | 1±8 | − | − | − | 0.7±1.2 | 3±7 | − | − | − | 0.6±1 | − |
| 23 | Iran | 36 | ICP-OES | − | − | − | 0.008±0.005 | 0.02±0.02 | 0.09±0.09 | − | 0.005±0.009 | − | − | − |
| 24* | Serbia | 5 | − | − | − | − | 0.1±0.0009 | 0.09±0.007 | − | − | 0.004±0.0005 | − | − | − |
| 25* | Iran | - | LIBS | 8.9 | − | − | 10.6± 0.32 | 0.0008 | 28.1±8.32 | − | 0.0006 | − | − | 0.0008 |
| 26* | China | 8 | ICP-MS | − | − | − | − | 0.01±0.0008 | − | − | 0.003±0.0003 | − | − | − |
| 27* | Iran | - | AAS | − | − | − | 0.6±0.1 | 0.003±0.0019 | 5.06±0.39 | − | 0.00029±9×10^-5^ | − | − | − |
| 28 | Iran | 15 | ICP-SFMS | − | 0.13±0.08 | − | − | 0.01±0.007 | − | 0.02±0.009 | 0.0039±0.002 | − | − | 0.002±0.001 |
| 29 | Iran | 50 | − | − | − | − | 0.3±0.1 | 0.009± 0.001 | 0.4±0.1 | − | 0.001± 0.00049 | − | − | − |
| 30 | Slovakia | - | AAS | 1.9±1.4 | − | 1.01± 0.3 | 0.9±0.2 | − | 11.3±1.9 | − | 0.3± 0.2 | − | − | − |
| 31* | South Korea | 64 | ICP-MS | − | − | 0.1± 0.0002 | 0.3± 0.0005 | 0.01±3.8×10^-5^ | 4.7± 0.006 | − | 0.002±2.3×10^-5^ | 0.005±1.5×10^-5^ | 0.3±0.0004 | 0.0019±6.8×10^-5^ |
| 32 | Egypt | 10 | AAS | − | 1.8±0.07 | − | − | 0.7±0.04 | − | − | 0.3±0.01 | − | − | − |
| 33 | Romania | 8 | ICP-MS | − | − | 0.001±0.0005 | 0.2±0.08 | 0.01±0.008 | 3.2±1.04 | − | 0.01±0.005 | 0.001±0.0001 | 0.06±0.05 | − |
| 34* | Brazil | − | ASV | − | − | − | − | 0.2± 0.09 | 13.08± 0.4 | − | 0.07± 0.005 | − | − | − |
| 35 | Pakistan | − | AAS | 0.2 ± 0.1 | − | 0.006± 0.001 | 0.4 ± 0.5 | 0.0006± 0.001 | 1.9± 1.7 | − | 0.03± 0.02 | − | 0.0001±0.001 | − |
| 36* | Romania | − | ICP-AES | 11.8 | 4.16 | 0.05 | 0.1 | 11×10-5 | 1.5 | − | 5×10-6 | − | 0.1 | − |
| 37* | Egypt | 60 | AAS | 0.6±0.4 | − | 0.03±0.02 | 0.1±0.2 | 0.02±0.02 | 3.1±0.6 | − | 0.02±0.02 | 0.03±0.02 | 0.03±0.02 | − |
| 38* | Poland | − | AAS | 4.1± 0.2 | − | − | − | − | 3.79± 0.06 | − | − | − | − | − |
| 39 | Pakistan | 60 | ET-AAS | − | 1.8±0.13 | **0.2±0.01** | − | 0.05±0.004 | − | − | 0.05±0.002 | − | − | − |
| 40* | China | 32 | ICP-OES | 2.4±0.59 | − | − | 0.3±0.05 | 0.03±0.008 | 3.6±1.66 | ND | 0.004±0.003 | − | 0.1±0.05 | ND |
| 41* | Pakistan | − | − | − | − | − | 0.2±0.004 | − | − | − | − | − | ND | − |
| 42 | Brazil | 54 | FAAS | 1.05±0.89 | − | 0.07±0.07 | 1.7±0.8 | 0.2±0.1 | 4.59±1.3 | − | − | − | 0.079±0.05 | − |
| 43* | Turkey | − | Metrohm 746 VA trace | − | − | − | 0.09±0.0001 | 2.9± 0.05 | − | − | 0.6±0.0001 | − | − | − |
| 44 | Spain | − | ICP-MS | 0.18±0.01 | 0.03±0.004 | 0.01±0.001 | 0.02±0.003 | 0.002±0.0003 | 3.1± 0.2 | ND | 0.0005±0.00019 | − | 0.001± 0.0003 | − |
| 45* | Spain | 21 | ET-AAS | − | 1.7±0.5 | 0.07±0.03 | 0.1±0.05 | 0.079±0.07 | 0.7±0.2 | − | 0.006±0.004 | − | 0.05±0.05 | − |
| 46* | India | 75 | − | − | − | − | 0.1± 0.001 | 0.002± 0.001 | 4.2± 0.001 | − | 9×10^-5^ ± 0.002 | − | − | − |
| 47 | Islands | 18 | AAS | 0.17±0.02 | − | − | 0.1± 0.039 | − | 3.06± 0.1 | − | − | − | − | − |
| 48 | Spain | 10 | AAS | 0.3±0.03 | − | − | 0.1±0.01 | − | 3.6±0.06 | − | − | − | − | − |

Red color: level of metals above standard limit of international standards (International Dairy Federation, FAO/WHO, and Codex Alimentarius); green color: level of metals above standard limit of local Standards; level of As only above of China Food and Drug Administration (CFDA). AAS: Atomic Absorption Spectroscopy, MP-AES: Microwave Plasma - Atomic Emission Spectrophotometer, ICP-AES: Inductively Coupled Plasma Atomic Emission Spectroscopy, GFAAS: Graphite Furnace Atomic Absorption Spectroscopy, FAAS: Flame Atomic Absorption Spectrophotometer, ICP-MS: Inductively Coupled Plasma-Mass Spectrometry, ICP-OES: Inductively Coupled Plasma - Optical Emission Spectrometry, LIBS: Laser Induced Breakdown Spectroscopy, ICP-SFMS: Inductively Coupled Plasma Sector Field Mass Spectrometry, ASV: Anodic Stripping Voltammetry, ET-AAS: Electrothermal Atomic Absorption Spectrophotometer, −: no data available, ND: not detected N: sample size, * the maximum values is reported, SD: standard deviation

## 3.3 Heavy metal concentrations in pasteurized and sterilized milk

### 3.3.1. Pb

Lead is not only non-essential for living organisms but also has toxic properties, and its accumulation in the human body causes serious consequences for humam health (61, 62). Infant and children are vulnerable groups to Pb poisoning because childeren absorb four to five times as much ingested Pb as adults from pathways(63, 64). High concentraten of exposure Pb damages the central nervous system and brain, causing convulsions, and coma, intellectual disability, and even death(65, 66).

The standard Pb limit in milk is 0.02 mg/l set by Codex Alimentarius Commission and FAO/WHO standard, and EU(67, 68). In this systematic review, the mean Pb levels in pasteurized and sterilized milk samples are analyzed in 41 studies (85.5% of total studies) from 13 countries Pakistan(4, 7, 35, 48, 51), Libya(15, 28), Iran(3, 5, 8, 14, 17, 18, 25, 29, 34, 36, 41, 43, 55), Egypt(12, 33, 38, 46, 49), Indonesia(26), Brazil(16, 39, 47, 57), China(30, 37, 42), Serbia(40), North Korea(45), Romania(19, 31), Turkey(52), Spain(58, 59), and India(53) from 1999 to 2021(Table 3).

The average concentration of Pb in pasteurized and sterilized milk samples across globe ranged between 11×10^-5^(19) and 3.5 mg/ml(16). Lead was the most common heavy metal in pasteurized and sterilized milk samples found above permissible limits (0.02 mg/l)(67, 68) in 19(46.34%) studies were conducted in Libya(15), Iran(8, 36, 55), Egypt(12, 33, 38, 46), Pakistan(4, 35, 51), Brazil(16, 39, 47, 57), Serbia(40), Turkey(52), China(30) and Spain(59).

The highest Level of Pb levels in milk samples were detected in Brazil(3.5±3.3 mg/L)(16)and Egypt(3.5±1.5 mg/L)(38). In these studies, the high Pb concentrations were observed in milk samples obtained from farm located in contaminated areas(16, 38). Similarly, a high Pb level (2.9±0.05) was determined in Turkey(52). This mainly results from atmospheric pollution, excessive use of fertilizers and pesticides, contamination during production and packaging processes(52).

In Egypt, Eman Abdelftah et al., (2019) observed a high Pb content (1.9±0.2 mg/L) in milk samples which exposed to Pb through contaminated water or wastewater for agricultural purposes(33). This contaminated water may be directly used by livestock, especially dairy animals, through drinking water or by bioaccumulation in soil, fodder, and vegetables(33). Furthermore, high levels of Pb were obtained from sterilized milk samples collected from Brazil(0.7±1.2 mg/L)(39) and Egypt (0.7±0.04 mg/L)(46). These studies reported that industrial activities, using contaminated feed, and the use of Pb-containing conserved cans are main sources of Pb(39, 46).

In Egypt, Mostafa et al. (2018) presented evidence that a high Pb concentration (0.4±0.1 mg/L) measured in pasteurized milk might be due to environmental contamination with this metal(12). Suturovic´ et al. (2014) measured an average Pb concentration (0.09±0.007 mg/L) in Serbia, where the maximum standard Pb concentration in milk is 0.1 mg/kg. Their determined Pb concentrations in two pasteurized milk samples were close to the maximum permissible limit(40).

A elevated level of Pb in pasteurized milk samples was also reported in Spain(0.079±0.07 mg/L), which might result from contamination during the pasteurization process in the factory or during packaging(59). A higher elevated Pb concentration was also recorded in three studies from Libya and Pakistan(4, 15, 51). They reported that high concentration of Pb in all milk samples mainly results from environmental pollution in these areas such as atmospheric deposition, waste disposal, vehicle smoke, and urban wastewater, etc.(4, 15, 51).

Two studies were conducted in Iran reported that Pb concentration in 33% of milk samples (0.02±0.02 mg/L) were higher than permission level of WHO/FAO in all milk samples (Feizi et al. 2020; Sobhanardakani 2014) because all milk samples were collected from animal farms close to a lead-zinc smelting plant, mining waste storage, thermal power plants, and roads(8, 55).

The results of review highlighted that pasteurized and sterilized milk can a common source of Pb poisoning. The level of Pb in most of samples (70% to 100%) from developing countires(Iran, Egypt, Pakistan, Serbia, and Turkey) were beyond standard limit, less Pb contamination was found in milk samples from developed countries. This result mainly due to stricter implementation of regulations, accurate detection techniques, and controlled industrialization in developed countries compered with developing countries.

### 3.3.2. Cd

Cadmium (Cd) is one of the non-degradable highly toxic element with long half-life (15–30 years)(62, 66). This metal enters the environment through industrial activities, using chemical fertilizers, pesticides, vehicle traffic, coal combustion, and using Ni-Cd rechargeable batteries(69, 70). Cadmium first binds to blood cells and albumin and accumulates in tissues, especially kidneys(64), liver (15%), and muscle (20%)(69). The Itai Itai disease, hepatic, carcinogenesis, teratogenic, nephrotoxic, skeletal, and reproductive effects are detected as the multifaceted deleterious effects of this metal on the human health(63, 71). The International Agency for Research on Cancer (IARC) has classified Cd in group 1 carcinogens for humans(66).

In this systematic review, we retrieved 35(73%) studies that detected Cd in pasteurized and sterilized milk samples collected in 14 countries Pakistan(4, 7, 35, 48, 51), Iran(3, 8, 14, 17, 18, 29, 34, 36, 41, 43, 55), Iraq(32), Egypt(12, 38, 46, 49), Libya(28), China(30, 37, 42), Serbia(40), Slovakia(44), North Korea(45), Romania(19, 31), Brazil(47), Turkey(52), Spain(58, 59), and India(53) from 1999 to 2021(Table 3).

The average level of Cd in pasteurized and sterilized milk samples across globe ranged between 5×10^−6^(19) and 0.6mg/L(52)(Table3). It should be noted that the Cd concentraten in milk samples in 23 studies (65.7%) that was conductd in different countries Iran(8, 18, 29, 34, 36, 55), Iraqi(32), Libya(28),China(30, 42), Serbia(40), Brazil(47), Egypt(12, 38, 46, 49), Slovakia(44), Pakistan(4, 35, 48, 51), Turkey(52) and Spain(59) were above standard limit of the local standrad as well as international standards such as International Dairy Federation )0.0026 μg/g)(72), FAO/WHO and Codex Alimentarius(0.01 mg/L)(73).

This result can due to uncontrolled and rapid industrial development in these countries cause elevated level of Cd in food stuffs. In contrast, Data extracted from other studies (12 of 35 studies) were conducted in Pakistan(7), Iran(3, 14, 17, 41, 43), China(37), South Korea(45), Romania(19, 31), Spain(58) and India(53)(Table 3) showed minimum or no contamination of Cd above standard limit suggesting the suitable implementation of regulations in these countries.

From literature, highest Cd concentration(0.6±0.0001 mg/L) in milk samples was detected in Turkey(52). The authors concluded that the high Cd content in the consumed raw milk was caused by unwanted contamination during the production process, excessive use of chemical fertilizers and pesticides, and contact with employed equipment (e.g., mechanical milkers, metal containers, etc.). Consequently, such elements as Cd present in the equipment or containers could enter raw milk during the pasteurization and sterilization process(52). In their study, the measured Cd level exceeds the maximum Cd limit (0.01 mg/L) both in terms of the local standard, the FAO/WHO standard, and Codex Alimentarius(52).

In Slovakia, average of Cd level in sterilized milk samples was of 0.3±0.2 mg/L (Lukáčová et al. 2012), indicating that Cd concentration in milk samples exceeded the local standard limit(44). In Egypt, Salah Fathy Aal et al. (2012) claimed that a elevated level of Cd (0.3±0.01 mg/L) in sterilized milk samples, which was caused by Sn and Cd migration from milk packaging cans during the storage process(46). Similarly, Sahar Issa et al. (2015) announced a high Cd concentration (0.29±0.1 mg/L) in pasteurized milk in Egypt, which exceeded the maximum standard limit and animal feed contamination with Cd was known as the main source(38).

In Iran, Cd level in pasteurized milk were ranged from 0.005±0.009(55) mg/L to 0.02±0.04 mg/L(8), and almost 70% of the total milk samples contained highest Cd concentration higher than the permissible standard limit(8). They reported the highest Cd level was measured in milk samples collected from caws reared close to industerial areas, mine tailings, and areas with a high traffic volume can lead to Cd accumulation in livestock tissues and, particularly, in milk(8). Besides, equipment used in the production and packaging process is the likely other source of contamination(55).

Subsequently, Cd level of 0.05±0.002 mg/L in pasteurized milk was determined in Pakistan (Gul Kazi et al. 2006). The contamination of containers and equipment used in milking and pasteurization processes and environmental contamination were accounted to be among the factors affecting the elevated Cd concentration in the collected milk samples in their study(51).

### 3.3.3. As

Arsenic enters the environment and water through natural and anthropogenic sources, such as tanning, ceramic, and mining industries, chemical fertilizers, and pesticides. The dissolution of As-containing mineral rocks in nature and soils naturally containing high As contents can result in its entry into the water(61, 74, 75). Arsenic has been classified in group 1 of carcinogenic compounds by the International Agency for Research on Cancer (IARC) and EPA(76). After entry into the body, this metal is absorbed into the bloodstream and attacks vital organs(61). Short-term exposure to As may cause nausea and vomiting, reduced production of erythrocytes, abnormal heartbeat, irritation in hands and feet, and damage to blood vessels. Long-term exposure can produce skin lesions, various cancers, neurological problems, lung diseases, hypertension, cardiovascular diseases, infertility, miscarriage, and diabetes(77-79).

The present systematic review (Table 3) recorded As concentration measurements in pasteurized and sterilized milk samples in five (10.4%) out of 48 surveys in three countries Turkey(27), Iran(29, 34, 41), and North Korea(45) on the globe.

The average level of As in pasteurized and sterilized milk samples across globe ranged between 0.0008(41) and 0.007 mg/L(34). The maximum acceptable As level in milk has been specified at 0.1 mg/L by EU and FAO/WHO standards(80). In these five studies, As levels are in the safe range and were not reported beyond the local standard limits of the studied countries and EU and FAO/WHO standards. However, the number studies related to measuring As in milk samples across the world was so limited .

### 3.3.4. Hg

Mercury residue enters the water through mining, different factories including papermaking plants, using fungicides, waste burning, and industrial/domestic sewage(81). This toxic element is found in aqueous environments in the forms of metallic Hg, inorganic salt, and organic compounds with different toxicities(82). Mercury organic compounds are more toxic than its inorganic compounds(61, 83). The entry of the most toxic Hg form (methylmercury) into the human body causes minamata disease, which results in various neurological complications and even death(71). The highest inorganic Hg concentration is accumulated in kidneys whereas organic Hg is more inclined to brain cells(61).

The greater Hg concentration in milk than the standard limit can be attributed to the anthropogenic contamination of the environment with Hg, Hg mining, different factories including papermaking plants, overuse of pesticides, waste burning, and industrial/urban effluents(10, 70, 81). Consequently, this toxic element enters the bodies of lactating animals by using contaminated fodder and water(70, 84). Other sources of contamination with Hg in animal diets include fish meal used in animal feed(10, 13).

Mercury concentration in pasteurized and sterilized milk samples are measured in 2 studies from 2 countries (Brazil and Iran) from 2013 to 2015(16, 29). Data presented in table 2 showed that limited data are available related to the prevalence of Hg in pasteurized and sterilized milk. Of 4 articles studied on Hg, in pasteurized and sterilized milk samples, Hg were detected in two studies conducted in Iran and Brazil(16, 29), with an average Hg concentration of 0.02±0.009 (29) and 6.8±3.2(16), respectively.

A higher elevated Hg concentration (6.8±3.2 mg/L) than the FAO/WHO standard limit (0.01 mg/L)(85) was only reported in Brazil by Marcio Augusto et al. (2015)(16).

### 3.3.5. Cr

Chromium (Cr) occurs in the environment in two forms: trivalent chromium(III), it is natural and an essential nutrient occuring from litoghneic sources (e.g. plantes, animal, rocks, and volcanic gases), and hexavalent chromium (VI), which, is most commonly produced by anthropogenic activities(e.g. industrial sewage discharge, fertilizers, aircraft manufacturing, pesticides, some dyes, and the sewage of plating industries(83, 86). The IARC reported that Cr (VI) is much more toxic than Cr (III), for both chronic and acute exposures(87, 88). It was evidenced that the respiratory tract is the main target organ for Cr(VI) toxicity such as coughing, shortness, inhalation exposures, heezing, perforations, bronchitis, decreased pulmonary function, and pneumonia(89, 90).

Likewise, IARC have clearly noted that inhaled CR(VI) is a carcinogen element resulting in an
increased lung cancer via inhalation exposure(88, 91). Environmental Protection Agency (EPA) classified Cr (III) compounds as a non-carcinogenic element (group D), and no information are available on the reproductive or developmental effects of Cr (III) in human(92). Based on EPA, the reference concentration for Cr (VI) is 0.0001 and 0.000008 mg/m^3^ based on respiratory effects in rats and human, respectively(92).

In this review, the level of Cr in pasteurized and sterilized milk samples were measured in 16(33%) out of 48 studies in 10 countries Libya(15), Iraq(32), Egypt(12, 49), Turkey(27), Pakistan(4, 48), China(30, 37), Brazil(39, 57), North Korea(45), Romania(19, 31), and Spain(58, 59) across the world(Table 3).

The average level of Cr in pasteurized and sterilized milk samples across globe ranged between 0.0001(48) and 0.6 mg/L(39). In Brazil, a high Cr concentration(0.6±1) was reported in sterilized milk samples (Santos et al. 2014), which exceeded the maximum acceptable Brazilian standard(0.1 mg/L)(93).

The Cr contamination in milk samples was claimed to probably result from mines, using agricultural chemicals, diet enrichment of lactating cows with Cr-containing salts to compensate for mineral deficiencies, and Cr contamination during milk processing(39).

### 3.3.6. Zn

Zinc is essential for growth and development and plays a vital role in gene expression regulation, cell division, immune system function, and sexual maturity(94). Zn is naturally found in soil but its concentration increases unnaturally because of anthropogenic activities mines, coal, and waste burning. Pesticides and animal manure are the other sources of contamination with this metal(71). The main clinical symptoms of severe Zn deficiency in humans are growth retardation and delay in sexual and skeletal maturation(82, 95, 96). This element is daily needed for the body at different concentrations, but it causes toxic effects on human health at concentrations higher than the allowable limit. The excessive use of Zn impairs Cu absorption and potentially leads to Cu deficiency(83). There are few reports of acute Zn poisoning. Its demonstrations include nausea, vomiting, diarrhea, fever, lethargy, anemia, fatigue, and immune dysfunction(82, 96, 97).

Here, 32 (66.6%) studies measured Zn concentrations in pasteurized and sterilized milk samples in 15 countries Libya(15), Iran(8, 14, 17, 18, 41, 43, 55), Iraq(32), Egypt(12, 38, 49), Turkey(27), Pakistan(4, 35, 48), Brazil(16, 39, 47, 57), China(30, 37), Slovakia(44), North Korea(45), Romania(19, 31), Polland(50), Spain(54, 58, 59), India(53), and Island(60) from 1993 to 2020.

The average level of Zn in milk samples across globe ranged between 0.09(55) and 28.1 mg/L(41). Review on Zn concentration in Milk samples showed that 20 studies out of 32 (62.5%) found exceed permission level recommended by of WHO/FAO (2.5-6.7 mg/L)(96).

A higher elevated concentration was recorded in Iran (28.1±8.32 mg/L)(41), Egypt (7.7±2.9 mg/L)(38), Iraq (12.8 mg/L)(32), Slovakia (11.3±1.9)(44), and Brazil (13.08±0.4 mg/L)(47).

### 3.3.7. Ni

Nickel (Ni) is an essential mineral for humans and act as a cofactor for some enzymes(70, 97). Likewise, it is used industrial application such as steel industries and combustion of coal(61, 64). This metal has toxic effect at concentrations higher than the maximum permissible limits and causes cell damage and changes in the activity of enzymes and hormones(61, 71, 83). Long-term exposure damages different body organs and causes allergic skin reactions(64).

Table 2 represents the analyzed data extracted from 16(33%) out of 48 studies measured Ni in 10 countries Libya(15), Egypt(12, 49), Turkey(27), Pakistan(35, 48, 51), Brazil(16, 57), China(37), Slovakia(44), North Korea(45), Romania(19, 31) and Spain(58, 59) from 1999 to 2020.

Mean Ni concentration in pasteurized and sterilized milk samples ranges from 0.001(31) to 1.01 mg/L(44). According to the EU standard, the maximum acceptable limit for Ni has been determined at 0.2 mg/L(98).

A higher elevated concentration than permission limits was recorded in 4(25%) out of 16 studies from Slovakia (1.01±0.3 mg/L)(44), Pakistan (0.14±0.04-0.2±0.01 mg/L)(35, 51), and Brazil(0.15±0.3)(16), which was attributed to the use of Ni-containing vessels during transportation processes(44, 51).

### 3.3.8. Co

Cobalt (Co) is an essential element for the human, which exist in organic and inorganic forms. It acts as a an essential constituent of the B12 vitamin that plays a vital role in the formation of amino acids and some proteins in neurons, which are necessary for the proper function of the body(97). Exposure to Co is often mixed anthropogenic activities, including the use of Co-containing sludge, phosphate fertilizers, and activities such as mining, smelting, purification, and combustion of metals(71). This element does not accumulate in the body and is rapidly excreted through the urine as well as via feces and bile to a lesser extent(99). Its acute effects include congestion, edema, reduced pulmonary ventilation during inhalation, and pulmonary hemorrhage during inhalation(100, 101). The chronic inhalation of Co also causes asthma, respiratory irritation, decreased pulmonary function, pneumonia, cardiac effects, vomiting and nausea, diarrhea, liver disorders, and sensitive skin(102, 103).

In this study, six(12.5%) of 48 reviewed articles measured Co concentrations in pasteurized and sterilized milk samples in the Pakistan(35), Brazil(16), China(37), North Korea(45), Romania(31), and Egypt(49)(Table 3) from 2007 to 2015.

limited studies examined Co concentration in milk samples. The average level of Co in pasteurized and sterilized milk samples across globe ranged between 0.001±0.0001(31) and 1.05±0.8 mg/L(16). In these six studies, the Co concentration was in the immune range and was not reported beyond maximum permission level limit.

### 3.3.9. Fe

Iron is an essential element required by the human body, which is involved in important functions, including oxygen transfer in erythrocytes, helping in the correct function of enzymes, and immunity system enhancement; it also participates in DNA synthesis and electron transfer(70, 97, 104). Iron is released by natural processes such as soil erosion, volcanic activities, and anthropogenic activities including pesticide application, mining, metal industries., fossil fuels, industrial residues, and sewage(94). Since Fe can produce free radicals, its excessive concentrations can cause tissue damage and increase the risk of cancer development(65, 70, 97, 104).

Here, 19 studies (39.58%) measured Fe concentrations in pasteurized and sterilized milk were measured in 13 countries Libya(15), Iraq(32), Turkey(27), Pakistan(4, 35, 48), Iran(34, 41), Brazil(16, 39, 57), Slovakia(44), Romania(19), Egypt(49), Polland(50), China(30), Spain(54, 58) and Iceland(60) from 1993 to 2020.

The average level of Fe in pasteurized and sterilized milk samples across globe ranged between 0.17±0.02(60) and 28.1 mg/L(19). Out of 19 studies, Fe concentrations were higher than the local standard limits only in three studies(16%) were conducted Turkey(27), Egypt(49), and China(30), and Fe concentrations were lower than the local standard limits in 16 (84%)(4, 15, 16, 19, 32, 34, 35, 39, 41, 44, 48, 50, 54, 57, 58, 60).

Fatma Esra Totan, et al (2017) and Alou Arab et al. (2007) reported that high concentration of Fe examined pasteurized and sterilized milk samples could be due to use of iron containers for milk transportation and preservation(27, 49). Li-Qiang et al. claimed that the use of equipment with iron material for transportation and preservation of pasteurization milk could be the main pathway to increase concentration of Fe in milk samples(30).

### 3.3.10. Al

Currently, there is considerable evidence that Al may produce side effects(70). The Fe-binding protein, called transferrin, is the main carrier of Al ions in the plasma. After absorption, Al is distributed in all body tissues, but it further accumulates in some tissues, particularly bones and lungs(105). About 95% of Al is excreted through urine and a small amount via bile and feces(65). The symptoms indicative of higher Al content in the human body include nausea, mouth ulcers, skin ulcers, vomiting, and diarrhea; however, these symptoms are reported to occur slightly in the short term. Al also adversely affects the nervous system, leading to memory loss, balance problems, and diseases such as Alzheimer's disease and Parkinson's dementia(65, 71).

As shown in Table 3, Al content in pasteurized and sterilized milk samples was only studied in 10 (20.83%) out of 48 studies in seven countries Turkey(27), Pakistan(4, 51), China(37), Egypt(38, 46), Iran(29), Romani(19), and Spain(58, 59) from 1999 to 2017.

The average level of Al in pasteurized and sterilized milk samples across globe ranged between 0.03(58) and 3.35mg/L(4). According our results, Al were higher than standards limits only in three (30%) studies were conducted in Pakistan (3.35 mg/L)(4), Egypt (1.8±0.07)(46), and Pakistan(1.8±0.13)(51).

The highest Al level (3.35 mg/L) in milk samples was observed in Pakistan (Ahmed et al. 2016), which was attributed to fresh milk contamination in aluminum containers during the transportation process(4). It was evidenced that high concentration of Al result from the nature of using cans for pasteurized milk packaging(4, 46, 51). Metal alloys (e.g., Al, Fe, Ni, and some other metals) used for manufacturing tools and containers may be released into milk during the pasteurization process(4). Another reason is that Al may enter milk through milking equipment because of raw milk contact with the equipment used for the pasteurization process. The levels of some heavy metals (e.g., Al) may also increase in milk samples due to environmental contamination(46, 51).

### 3.3.11. Cu

Cu is an essential mineral for the normal growth of humans(82, 97). Acute toxicity with Cu is associated with symptoms such as abdominal pain, nausea, vomiting, diarrhea, gastrointestinal bleeding, headache, dizziness, fever and chills, muscle pain, hemolysis of erythrocytes, anemia, liver/kidney/muscle/skeletal/ nervous problems, shock, and death(95, 106).

According to this review(Table 3), Cu concentrations in pasteurized and sterilized milk samples were measured in 34 (70.83%) out of 48 studies in 15 countries Libya(15), Iran(8, 14, 17, 18, 34, 41, 43, 55), Iraq(32), Egypt(12, 33, 38, 49), Turkey(27, 52), Pakistan(4, 35, 48, 56), Brazil(16, 57), China(30, 37), Serbia(40), Slovakia(44), North Korea(45), Romania(19, 31), Spain(54, 58, 59), India(53), Island(60) from 1993 to 2020.

The average level of Cu in pasteurized and sterilized milk samples across globe ranged between 0.004(27) and 10.6 mg/L(41). It should be noted that 32 studies out of 34(94%) found to exceed the maximum limit of Cu in milk samples recommended by Standardization Administration of the People’s Republic of China (0.01 mg/L)(107) and WHO/FAO (0.05 mg/L)(108). A higher elevated concentration was recorded in Iran(10.6±5.32 mg/L)(41), Egypt (1.9±0.7 mg/L)(38), and Brazil (1.7±0.8 mg/L)(57).

The high Cu concentration was attributed to environmental contamination(17, 30, 38). Conversely, much lower level of Cu (Cu< 0.01 mg/l) were detected in only 2 studies were conducted in Iran and Turkey in 2014(27, 55).

Based on result of this review, about the prevalence of heavy metals in the pasteurized and sterilized milk samples, it is clear that Cu, Pb, Zn, and Cd were the predominant heavy metal among all the milk samples. This may be due to the way of these heavy metals excretion in milk samples depending quality of raw milk, manufacturing procedures, as well as heavy metal’s non-biodegradable properties and persistent nature after being accumulated and absorbed(9, 11, 109).

High concentration of heavy metals in raw milk may be attributed to contaminated animal feed and drinking water. Likewise, the mal-usage of fertilizers can affect the heavy metals uptake by the plants, which are consumed by the animals(109-112).

It is evidenced that the evaporation of milk during pasteurization process generally increase contaminant concentration in milk and its products. Furthermore, heavy metals including, Cd, Cu Pb, and Cr, preferentially bound to milk caseins, and improper production methods in unhygienic atmospheric contamianted conditions and poor packaging can transfer heavy metals to milk and its products(95, 113, 114).

## 3.4. Risk Assessment of pasteurized and sterilized milk consumption

### 3.4.1. Estimated daily intake (EDI)

Dietary exposure to Fe, Al, Ni, Cu, Pb, Zn, Hg, Cd, Co, Cr and As through pasteurized and sterilized milk consuming was evaluated by calculating EDI of these metals based on average of heavy metals in this review (Table 4). We compared value of EDI with the provisional tolerable daily intake (PTDI) to appraise the risk associated to the ingestion of heavy metal in milk.

According our finding, some studies reported that the EDI values of Pb(16, 38, 39, 52), Cd(44, 45, 52) and Hg(16), higher than PTDI limits, while others trace elements such as Fe, Al, Ni, Cu, Zn, Co, Cr, and As for all examined samples did not exceed the PTDI limits.

The Joint FAO/WHO Expert Committee on Food Additives recommended the provisional tolerable intakes of Pb as(0.0036 mg/kg BW/day)(68). Daily intake for the exposure of Pb through Pasteurized and sterilized milk consumption around the world ranged from 4.29E-07(28) to 1.99E-02(52)(mg/kg BW/day) which represent 0.012%–568.16% of PTDI(28, 52).

It should be noted that mean Pb uptake by milk consumption in 4(8.33%) out of 48 studies across the globe were extremely high and it was exceeded 100% of PTDI(16, 38, 39, 52). The average consumption of Pb through milk reaches its maximum in milk collected in Turkey(52), Brazil(16, 39) and Egypt(38), the values were 568.16%, 557.14%, 111.42% and 157.14% of PTDI respectively(Table 4), which indicated the high potential health risk from Pb tested upon consumption in these milk samples. In 16 (39.02%) studies the values were between 1% to 93% of PTDI. In 21 studies (51.21%) were representing values <1% of PTDI.

For Cd, the PTMI is 0.00083 mg/kg BW/day (68). Cadmium uptake by milk consumption observed in 36 (75%) out of 48 studies. The exposure of Cd through Pasteurized and sterilized milk consumption across the globe ranged between 4.64E-08(19) to 4.11E-03(52) (mg/kg BW/day) which represented the values ranged from 0.0055%(19) to 495.69%(52)of PTDI. The average consumption of Cd through milk collected in Turkey (495.69%)(52) and Slovakia (222.03%)(44) covers maximum values and of PTDI. The average Cd consumption exceed 100% of PTDI in 2 Studies across the globe (44, 52), while the values ranged between 1% to 90% of PTDI in 18 Studies (51.42%), and 15 studies (42.85%) were representing values <1% of PTDI (Table 4).

Regarding Hg, a recommended PTDI of 0.00057 mg/kg BW/day have been set by the 72th JECFA(the Joint FAO/WHO Expert Committee on Food Additives) in 2010 form inorganic mercury(68). It should be noted that Hg uptake by milk consumption only observed in 2 (4.1%) out of 48 studies(16, 29). The average of Hg consumption through milk ranged between 4.0E-05 to 3.78E-02 (mg/kg BW/day) which represent 7.01% to 6646.61% of PTDI. The average consumption of Hg in sterilized milk collected from Brazil (6646.61%) covers maximum value of PTDI(16)(Table 4).

Therefore, it is seem that exposure to Pb(16, 38, 39, 52), Cd(44, 45, 52) and Hg(16), through milk consumption contributed the highest portion of EDI.

Table 4. Estimated Daily Intake (EDI) in (mg/kg BW/day) of heavy metals

| **code** | **EDI (mg/kg BW/day)** | | | | | | | | | | |
| --- | --- | --- | --- | --- | --- | --- | --- | --- | --- | --- | --- |
|  | **Fe** | **Al** | **Ni** | **Cu** | **Pb** | **Zn** | **Hg** | **Cd** | **Co** | **Cr** | **As** |
| 1 | − | − | − | − | 4.29E-05 | − | − | 1.43E-05 | - | - | - |
| 2 | 7.71E-05 | - | 3.0E-06 | 4.28E-06 | 2.14E-06 | 8.61E-05 | - | - | - | 1.71E-06 | - |
| 3 | - | - | - | 1.8E-03 | 4.00E-05 | 8.6E-03 | - | 4.00E-05 | - | - | - |
| 4 | - | - | - | - | 1.60E-05 |  | - | - | - | - | - |
| 5 | - | - | - | - | 2.00E-05 |  | - | - | - | - | - |
| 6 | 1.14E-03 | - | - | 5.71E-05 | - | 7.31E-03 | - | 2.28E-04 | - | 8.57E-04 | - |
| 7 | - | - | - | 9.43E-04 | 2.99E-03 |  | - | - | - | - | - |
| 8 | - | - | - | - | 6.00E-06 |  | - | 8.00E-07 | - | - | - |
| 9 | - | - | 1.10E-04 | 3.14E-04 | 6.29E-04 | 2.20E-03 | - | 4.71E-06 | - | 1.10E-04 | - |
| 10 | - | - | - | - | 1.03E-06 |  | - | - | - | - | - |
| 11 | 1.30E-02 | 4.11E-04 | 6.17E-05 | 2.74E-05 | - | 5.48E-03 | - | - | - | 6.85E-05 | 3.36E-05 |
| 12 | 1.41E-02 | 2.39E-02 | - | 3.5E-03 | 3.57E-04 | 1.92E-02 | - | 2.85E-05 | - | 6.42E-04 | - |
| 13 | 6.2E-03 | - | - | 4.0E-04 | 2.0E-05 |  | - | 1.96E-05 | - | - | 1.4E-05 |
| 14 | - | - | - | - | 4.29E-07 |  | - | 3.86E-06 | - | - | - |
| 15 | - | - | - | 4.0E-04 | 8.0E-05 | 1.80E-03 | - | 9.80E-05 | - | - | - |
| 16 | - | - | - | 6.0E-04 | 1.80E-05 | 8.00E-04 | - | 2.00E-06 | - | - | - |
| 17 | 1.14E-02 | - | 7.14E-04 | 1.42E-04 | 1.35E-03 | 4.85E-02 | - | 2.85E-04 | 3.57E-04 | - | - |
| 18 | - | - | - | - | 8.00E-05 |  | - | 1.20E-05 | - | - | - |
| 19 | 7.24E-03 | - | 5.57E-04 | 1.11E-03 | 1.95E-02 | 2.56E-02 | 3.78E-02 | - | 5.85E-03 | - | - |
| 20 | - | 8.57E-05 | 4.28E-05 | 3.42E-05 | 5.14E-06 | 2.4E-03 | - | 1.62E-06 | 2.57E-06 | 8.57E-06 | - |
| 21 | - | 2.2E-03 | - | 2.98E-03 | 5.5E-03 | 1.21E-02 | - | 4.55E-04 | - | - | - |
| 22 | 5.57E-03 | - | - | - | 3.9E-03 | 1.67E-02 | - | - | - | 3.34E-03 | - |
| 23 | - | - | - | 1.6E-05 | 4.00E-05 | 1.80E-04 | - | 1.00E-05 | - | - | - |
| 24 | - | - | - | 6.71E-04 | 6.04E-04 | - | - | 2.69E-05 | - | - | - |
| 25 | 1.78E-02 | - | - | 2.12E-02 | 1.6E-06 | 5.62E-02 | - | 1.38E-06 | - | - | 1.6E-06 |
| 26 | - | - | - | - | 9.29E-06 | - | - | 2.79E-06 | - | - | - |
| 27 | - | - | - | 1.20E-03 | 6.00E-06 | 1.01E-02 | - | 5.80E-07 | - | - | - |
| 28 | - | 2.0E-04 | - | - | 2.0E-05 | - | 4.0E-05 | 6.0E-06 | - | - | 4.0E-06 |
| 29 | - | - | - | 6.00E-04 | 1.80E-05 | 8.0E-04 | - | 2.0E-06 | - | - | - |
| 30 | 1.16E-02 | - | 6.2E-03 | 5.52E-03 | - | 6.99E-02 | - | 1.14E-03 | - | - | - |
| 31 | - | - | 5.71E-05 | 1.71E-04 | 5.71E-06 | 2.69E-03 | - | 1.84E-03 | 2.86E-06 | 1.71E-04 | 5.71E-07 |
| 32 | - | 2.82E-03 | - | - | 1.1E-03 | - | - | 4.71E-04 | - | - | - |
| 33 | - | - | 9.29E-06 | 1.86E-03 | 9.29E-05 | 2.97E-02 | - | 9.29E-05 | 9.29E-06 | 5.57E-04 | - |
| 34 | - | - | - | - | 1.11E-03 | 7.29E-02 | - | 3.90E-04 | - | - | - |
| 35 | 1.42E-03 | - | 4.28E-05 | 2.85E-03 | 4.28E-06 | 1.35E-02 | - | 2.14E-04 | - | 7.14E-07 | - |
| 36 | 1.09E-01 | 3.8E-02 | 4.64E-04 | 9.28E-04 | 9.28E-07 | 1.39E-02 | - | 4.64E-08 | - | 9.28E-04 | - |
| 37 | 9.42E-04 | - | 4.71E-05 | 1.57E-04 | 3.14E-05 | 4.87E-03 | - | 3.14E-05 | 4.71E-05 | 4.71E-05 | - |
| 38 | 2.81E-02 | - | - | - | - | 2.59E-02 | - | - | - | - | - |
| 39 | - | 1.28E-02 | 1.42E-03 | - | 3.57E-04 | - | - | 3.57E-04 | - | - | - |
| 40 | 2.05E-03 | - | - | 2.57E-04 | 2.57E-05 | 3.08E-03 | - | 3.42E-06 | - | 8.57E-05 | - |
| 41 | - | - | - | 1.43E-03 | - | - | - | - | - | - | - |
| 42 | 5.85E-03 | - | 3.9E-04 | 9.47E-03 | 1.11E-03 | 2.55E-02 | - | - | - | 4.40E-04 | - |
| 43 | - | - | - | 6.17E-04 | 1.99E-02 | - | - | 4.11E-03 | - | - | - |
| 44 | 7.0E-04 | 2.1E-04 | 7.0E-05 | 1.4E-04 | 1.4E-05 | 2.17E-02 | - | 3.5E-06 | - | 7.0E-06 | - |
| 45 | - | 1.19E-02 | 4.9E-04 | 7.0E-04 | 5.53E-04 | 4.9E-03 | - | 4.2E-05 | - | 3.5E-04 | - |
| 46 | - | - | - | 4.14E-04 | 8.29E-06 | 1.74E-02 | - | 3.73E-07 | - | - | - |
| 47 | 1.0E-03 | - | - | 1.0E-03 | - | 3.06E-02 | - | - | - | - | - |
| 48 | 2.1E-03 | - | - | 7.0E-04 | - | 2.52E-02 | - | - | - | - | - |

PTDI values: Fe(0.8 mg/kg BW/day); Al(0.14 mg/kg BW/day); Ni(0.3 mg/kg BW/day); Cu(0.5 mg/kg BW/day); Pb(0.0035mg/kg BW/day); Zn(1 mg/kg BW/day); Hg(0.00057 mg/kg BW/day) (for inorganic mercury); Cd(0.0008 mg/kg BW/day); Co(0.005 to 0.04); Cr(0.05 to 0.2 ); As(0.0021 mg/kg BW/day)(68, 115); –: no data available; The red color shows the EDI value above the PTDI values

### 3.4.2. Non-carcinogenic risk assessment

The non-carcinogenic risk of heavy metals for the milk consumers was determined by calculating the non-carcinogenic hazard quotient (HQ) and hazard index (HI) values (Table 5). If THQ of heavy metals is lower than the threshold of the US EPA criteria (HQ< 1), non-carciongenic risk are improbable to happen. Non-carcinogenic effect can occur to exposed population if THQ is higher than the threshold of the US EPA criteria (HQ> 1)(96).

In milk samples, the average value of HQ for most heavy metals were found to be lower than the threshold of the US EPA criteria, except for Pb(16, 38, 39, 52), Hg(16), Cd(44, 52)and Co(16).

Therefore, non-carcinogenic risk is expected for Pb, Hg, Co, and Cd. Review on THQ values showed that milk consumers in 4 studies out of 48 in the Brazil(16, 39), Egypt(38), Turkey(52) were exposed to high health risk through the intake of Pb.

The highest value of THQ for Pb was estimated to be 5.68E+00 in pasteurized and sterilized milk collected in Turkey(52). Also, THQ of Pb was more than one in milk samples collected from Brazil(from 1.11E+00 to 5.57E+00)(16, 39) and Egypt (1.57E+00)(38).

Review on the THQ of Cd showed that milk consumers in 2 studies was conducted in the Slovakia(44) and Turkey(52) were exposed to high health risk through the intake of Cd. The highest THQ value of Cd was 4.11E+00 recorded in pasteurized and sterilized milk collected in Turkey(52) in (2002). Also, THQ values were higher than 1 in sterilized milk collected in Slovakia (1.84E+00)(44) in 2012.

For Hg and Co, THQ values of milk samples exceeded 1 in only one study were conducted in Brazil(16). The results of non-carcinogenic risks from exposure to metals through milk consumption indicates that Pasteurized and sterilized milk collected in all sites during the last decade was safe for human consumption in terms of the amounts of Fe, Al, Cu, Ni, Zn, Cr and As (THQ values <1; Table 5).

Based on risk results, higher THQ value of>1 was detected for Pb, Cd, Co and Hg in pasteurized and sterilized milk samples examined in Slovakia(44), Brazil(16, 39), Turkey(52), and Egypt(38). It seem that milk consumer in these regions can be exposed to potential health risks upon milk consumption. Further attention must be paid to control contamination sources of these metals.

Table 5. Target hazard quotient (THQ) values for heavy metals in Pasteurized and sterilized milk reported in research articles published since 1993-2021.

| **code** | **THQ** | | | | | | | | | | | **TTHQ** |
| --- | --- | --- | --- | --- | --- | --- | --- | --- | --- | --- | --- | --- |
|  | **Fe** | **Al** | **Ni** | **Cu** | **Pb** | **Zn** | **Hg** | **Cd** | **Co** | **Cr** | **As** |  |
| 1 | - | - | - | - | 1.22E-02 | - | - | 1.43E-02 | - | - | - | 2.65E-02 |
| 2 | 1.10E-04 | - | 1.50E-05 | 1.07E-04 | 6.12E-04 | 2.87E-04 | - | - | - | 1.14E-06 | - | 1.13E-03 |
| 3 | - | - | - | 4.50E-02 | 1.14E-02 | 2.87E-02 | - | 4.00E-02 | - | - | - | 1.25E-01 |
| 4 | - | - | - | - | 4.57E-03 | - | - | - | - | - | - | 4.57E-03 |
| 5 | - | - | - | - | 5.71E-03 | - | - | - | - | - | - | 5.71E-03 |
| 6 | 1.64E-03 | - | - | 1.43E-03 | - | 2.44E-02 | - | 2.29E-01 | - | 5.71E-04 | - | 2.57E-01 |
| 7 | - | - | - | 2.36E-02 | 8.53E-01 | - | - | - | - | - | - | 8.77E-01 |
| 8 | - | - | - | - | 1.71E-03 | - | - | 8.00E-04 | - | - | - | 2.51E-03 |
| 9 | - | - | 5.50E-04 | 7.86E-03 | 1.80E-01 | 7.33E-03 | - | 4.71E-03 | - | 7.33E-05 | - | 2.00E-01 |
| 10 | - | - | - | - | 2.94E-04 | - | - | - | - | - | - | 2.94E-04 |
| 11 | 1.86E-02 | 5.88E-04 | 3.09E-04 | 6.86E-04 | - | 1.83E-02 | - | - | - | 4.57E-05 | 1.12E-01 | 1.51E-01 |
| 12 | 2.02E-02 | 3.42E-02 | - | 8.75E-02 | 1.02E-01 | 6.40E-02 | - | 2.86E-02 | - | 4.29E-04 | - | 3.37E-01 |
| 13 | 8.86E-03 | - | - | 1.00E-02 | 5.71E-03 | - | - | 1.96E-02 | - | - | 4.67E-02 | 9.08E-02 |
| 14 | - | - | - | - | 1.22E-04 | - | - | 3.86E-03 | - | - | - | 3.98E-03 |
| 15 | - | - | - | 1.00E-02 | 2.29E-02 | 6.00E-03 | - | 9.80E-02 | - | - | - | 1.37E-01 |
| 16 | - | - | - | 1.50E-02 | 5.14E-03 | 2.67E-03 | - | 2.00E-03 | - | - | - | 2.48E-02 |
| 17 | 1.63E-02 | - | 3.57E-03 | 3.57E-03 | 3.88E-01 | 1.62E-01 | - | 2.86E-01 | 1.00E-01 | - | - | 1.85E+00 |
| 18 | - | - | - | - | 2.29E-02 | - | - | 1.20E-02 | - | - | - | 3.49E-02 |
| 19 | 1.03E-02 | - | 2.79E-03 | 2.79E-02 | 5.57E+00 | 8.54E-02 | 9.47E+01 | - | 1.46E+01 | - | - | 1.15E+02 |
| 20 | - | 1.22E-04 | 2.14E-04 | 8.57E-04 | 1.47E-03 | 8.00E-03 | - | 1.63E-03 | 6.43E-03 | 5.71E-06 | - | 1.87E-02 |
| 21 | - | 3.14E-03 | - | 7.46E-02 | 1.57E+00 | 4.03E-02 | - | 4.56E-01 | - | - | - | 2.15E+00 |
| 22 | 7.96E-03 | - | - | - | 1.11E+00 | 5.57E-02 | - | - | - | 2.23E-03 | - | 1.18E+00 |
| 23 | - | - | - | 4.00E-04 | 1.14E-02 | 6.00E-04 | - | 1.00E-02 | - | - | - | 2.24E-02 |
| 24 | - | - | - | 1.68E-02 | 1.73E-01 | - | - | 2.69E-02 | - | - | - | 2.16E-01 |
| 25 | 2.54E-02 | - | - | 5.30E-01 | 4.57E-04 | 1.87E-01 | - | 1.38E-03 | - | - | 5.33E-03 | 7.50E-01 |
| 26 | - | - | - | - | 2.65E-03 | - | - | 2.79E-03 | - | - | - | 5.44E-03 |
| 27 | - | - | - | 3.00E-02 | 1.71E-03 | 3.37E-02 | - | 5.80E-04 | - | - | - | 6.60E-02 |
| 28 | - | 2.86E-04 | - | - | 5.71E-03 | - | 1.00E-01 | 6.00E-03 | - | - | 1.33E-02 | 1.25E-01 |
| 29 | - | - | - | 1.50E-02 | 5.14E-03 | 2.67E-03 | - | 2.00E-03 | - | - | - | 2.48E-02 |
| 30 | 1.67E-02 | - | 3.10E-02 | 1.38E-01 | - | 2.33E-01 | - | 1.84E+00 | - | - | - | 2.26E+00 |
| 31 | - | - | 2.86E-04 | 4.29E-03 | 1.63E-03 | 8.95E-03 | - | 1.14E-03 | 7.14E-03 | 1.14E-04 | 1.90E-03 | 2.55E+-2 |
| 32 | - | 4.04E-03 | - | - | 3.14E-01 | - | - | 4.71E-01 | - | - | - | 7.90E-01 |
| 33 | - | - | 4.64E-05 | 4.64E-02 | 2.65E-02 | 9.90E-02 | - | 9.29E-02 | 2.32E-02 | 3.71E-04 | - | 2.88E-01 |
| 34 | - | - | - | - | 3.18E-01 | 2.43E-01 | - | 3.90E-01 | - | - | - | 9.51E-01 |
| 35 | 2.04E-03 | - | 2.14E-04 | 7.14E-02 | 1.22E-03 | 4.52E-02 | - | 2.14E-01 | - | 4.76E-07 | - | 3.34E-01 |
| 36 | 1.57E-01 | 5.44E-02 | 2.32E-03 | 2.32E-02 | 2.65E-04 | 4.64E-02 | - | 4.64E-05 | - | 6.19E-04 | - | 2.84E-01 |
| 37 | 1.35E-03 | - | 2.36E-04 | 3.93E-03 | 8.98E-03 | 1.62E-02 | - | 3.14E-02 | 1.18E-01 | 3.14E-05 | - | 1.8E-01 |
| 38 | 4.02E-02 | - | - | - | - | 8.66E-02 | - | - | - | - | - | 1.27E-01 |
| 39 | - | 1.84E-02 | 7.14E-03 | - | 1.02E-01 | - | - | 3.57E-01 | - | - | - | 4.85E-01 |
| 40 | 2.94E-03 | - | - | 6.43E-03 | 7.35E-03 | 1.03E-02 | - | 3.43E-03 | - | 5.71E-05 | - | 3.05E-02 |
| 41 | - | - | - | 3.57E-02 | - | - | - | - | - | - | - | 3.57E-02 |
| 42 | 8.36E-03 | - | 1.95E-03 | 2.37E-01 | 3.18E-01 | 8.52E-02 | - | - | - | 2.93E-04 | - | 6.51E-01 |
| 43 | - | - | - | 1.54E-02 | 5.68E+00 | - | - | 4.11E+00 | - | - | - | 9.81E+00 |
| 44 | 1.00E-03 | 3.00E-04 | 3.50E-04 | 3.50E-03 | 4.00E-03 | 7.23E-02 | - | 3.50E-03 | - | 4.67E-06 | - | 8.50E-02 |
| 45 | - | 1.70E-02 | 2.45E-03 | 1.75E-02 | 1.58E-01 | 1.63E-02 | - | 4.20E-02 | - | 2.33E-04 | - | 2.12E-01 |
| 46 | - | - | - | 1.04E-02 | 2.37E-03 | 5.80E-02 | - | 3.73E-04 | - | - | - | 7.11E-02 |
| 47 | 1.43E-03 | - | - | 2.50E-02 | - | 1.02E-01 | - | - | - | - | - | 1.28E-01 |
| 48 | 3.00E-03 | - | - | 1.75E-02 | - | 8.40E-02 | - | - | - | - | - | 1.05E-01 |

–: no data available; The red color shows the THQ value above the recommended value of EPA.

### 3.4.3. Carcinogenic risk assessment of heavy metals

Table 6 represents the carcinogenic risk (CR) of heavy metals in pasteurized and sterilized milk samples in different countries around the world. According to EPA, if CR of metals is Less than 10^-6^, carcinogenic adverse effect are improbable to the exposed population. Carcinogenic adverse effect may happen to exposed population if CR higher than 10^-6^. Cancer risk was measured for 44 studies included in a systematic review,(Table 6).

Based on risk results, 11(25%)studies in Libya(15), Iran(3, 5, 14, 17, 25, 43), Indonesia(26), China(37), Romania(19) and India(53) indicated acceptable or negligible levels of heavy metals (CR <10 ^-6^) via consumption of milk samples, and 25(56.8%) studies reported moderate level of risk (10 ^-4^<CR ≤10 ^-6^) in Pakistan(4, 7, 48), Iran(8, 18, 29, 34, 36, 41, 55), Iraqi(32), Egypt(12, 33, 49), Turkey(27), Libya(28), Brazil(39, 57), Serbia(40), China(30, 42), South Korea(45), Romania(31) and Spain(58, 59).

Likewise, 8 included studies indicated high level of risk (CR≥10^-4^) via consumption of milk samples contaminated with heavy metals compound in Pakistan(35), Brazil(16), Egypt(38, 46), Slovakia(44), Brazil(47), Pakistan(51), Turkey(52).

Review on CR values showed that milk consumers in 14 studies (31.8%) out of 44 studies in the Egypt(12, 33, 38, 46), Pakistan(4, 35, 51), Brazil(16, 39, 47, 57), Serbia(40), Turkey(52) and Spain(59) were exposed to moderate or high carcinogenic risk through the intake of Pb. The highest value of cancer risk for Pb was measured to be 1.66E-04 and 1.69E-04 in pasteurized and sterilized milk collected from Brazil(16) and Turkey(52), respectively.

About Cd, 35 (73%) of 48 studies included in this review measured Cd concentration in milk samples. They reported milk consumer in 26 studies (74%) out of 35 studies in Pakistan(4, 7, 35, 48, 51), Iran(8, 18, 29, 34, 36, 55), Iraqi(32), Egypt(12, 38, 46, 49), Libya(28), Serbia(40), China(30, 42), Slovakia(44), Romania(31), Brazil(47), Turkey(52), and Spain(58, 59) were exposed to moderate to high carcinogenic risk through the intake of Cd. The highest value of cancer risk for Cd was 7.00E-04 in pasteurized and sterilized milk samples collected from Slovakia(44).

Arsenic and Hg, were reported only by 5 and 2 studies, respectively. They reported milk consumer in these studies were exposed to moderate carcinogenic risk through the intake of Hg and As. Although, limited studies measured As and Hg contamination in milk, these element could be a growing public concern in world.

The high exposure to heavy metals contamination especially Pb and Cd is certain public health concern, therefore, maximum control measures and stricter regulation must be adopted to chemical contaminants in the dairy industry, becuase that milk is the main products by humans worldwide.

Table 6. Carcinogenic risk assessment values for heavy metals in Pasteurized and sterilized milk reported in research articles published since 1993-2021.

| **code** | **CR** | | | | **TCR** |
| --- | --- | --- | --- | --- | --- |
|  | **As** | **Hg** | **Pb** | **cd** |  |
| 1 | - | - | 3.64E-07 | 5.43E-06 | 5.79E-06 |
| 2 | - | - | 1.82E-08 | - | 1.82E-08 |
| 3 | - | - | 3.40E-07 | 1.52E-05 | 1.55E-05 |
| 4 | - | - | 1.36E-07 | - | 1.36E-07 |
| 5 | - | - | 1.70E-07 | - | 1.70E-07 |
| 6 | - | - | - | 8.69E-05 | 8.69E-05 |
| 7 | - | - | 2.54E-05 | - | 2.54E-05 |
| 8 | - | - | 5.10E-08 | 3.04E-07 | 3.55E-07 |
| 9 | - | - | 5.34E-06 | 1.79E-06 | 7.13E-06 |
| 10 | - | - | 8.74E-09 | - | 8.74E-09 |
| 11 | 5.04E-05 | - | - | - | 5.04E-05 |
| 12 | - | - | 3.04E-06 | 1.09E-05 | 1.39E-05 |
| 13 | 2.10E-05 | - | 1.70E-07 | 7.45E-06 | 2.86E-05 |
| 14 | - | - | 3.64E-09 | 1.47E-06 | 1.47E-06 |
| 15 | - | - | 6.80E-07 | 3.72E-05 | 3.79E-05 |
| 16 | - | - | 1.53E-07 | 7.60E-07 | 9.13E-07 |
| 17 | - | - | 1.15E-05 | 1.09E-04 | 1.21E-04 |
| 18 | - | - | 6.80E-07 | 4.56E-06 | 5.24E-06 |
| 19 | - | 1.14E-05 | 1.66E-04 | - | 1.77E-04 |
| 20 | - | - | 4.37E-08 | 6.19E-07 | 6.63E-07 |
| 21 | - | - | 4.68E-05 | 1.73E-04 | 2.20E-04 |
| 22 | - | - | 3.32E-05 | - | 3.32E-05 |
| 23 | - | - | 3.40E-07 | 3.80E-06 | 4.14E-06 |
| 24 | - | - | 5.14E-06 | 1.02E-05 | 1.53E-05 |
| 25 | 2.40E-06 | - | 1.36E-08 | 5.24E-07 | 2.94E-06 |
| 26 | - | - | 7.89E-08 | 1.06E-06 | 1.14E-06 |
| 27 | - | - | 5.10E-08 | 2.20E-07 | 2.71E-07 |
| 28 | 6.00E-06 | 1.20E-08 | 1.70E-07 | 2.28E-06 | 8.46E-06 |
| 29 | - | - | 1.53E-07 | 7.60E-07 | 9.13E-07 |
| 30 | - | - | - | 7.00E-04 | 7.00E-04 |
| 31 | 8.57E-07 | - | 4.86E-08 | 4.34E-07 | 1.34E-06 |
| 32 | - | - | 9.35E-06 | 1.79E-04 | 1.88E-04 |
| 33 | - | - | 7.89E-07 | 3.53E-05 | 3.61E-05 |
| 34 | - | - | 9.47E-06 | 1.48E-04 | 1.57E-04 |
| 35 | - | - | 3.64E-08 | 8.14E-05 | 8.14E-05 |
| 36 | - | - | 7.89E-09 | 1.76E-08 | 2.55E-08 |
| 37 | - | - | 2.67E-07 | 1.19E-05 | 1.22E-05 |
| 39 | - | - | 3.04E-06 | 1.36E-04 | 1.39E-04 |
| 40 | - | - | 2.19E-07 | 1.30E-06 | 1.52E-06 |
| 42 | - | - | 9.47E-06 | - | 9.47E-06 |
| 43 | - | - | 1.69E-04 | 1.56E-03 | 1.73E-03 |
| 44 | - | - | 1.19E-07 | 1.33E-06 | 1.45E-06 |
| 45 | - | - | 4.70E-06 | 1.60E-05 | 2.07E-05 |
| 46 | - | - | 7.04E-08 | 1.42E-07 | 2.12E-07 |

–: no data available.

# Conclusion

This review is the first study that was conducted on the concentration and potential health risk of heavy metals in the pasteurized and sterilized milk recorded across the world. The elevated levels of all heavy metals, in milk samples, except of Co, As and Hg, calls for maximum control and strict regulation by environmental and health protection. Based on result of this review, Cu, Cd, Zn, and Pb were the most common contaminants, which exceeded the maximum permissible criteria in 94%, 67%, 62%, and 46% of the milk samples analysed, respectively followed by Al, Fe, and Ni, which exceeded the local maximum permissible criteria in 30%, 16%, and 12.5% of the milk tested, respectively. Arsenic, Co, and Hg showed the least contamination level and were lower than the maximum permissible limits recommended by local standards of the studies countries, FAO/WHO, Codex Alimentarius, and EU standards.

According to the health risk assessment results, since THQ results for Pb, Hg, Co and Cd were higher than one in some sample, indicated that milk consumers would be exposed to health risk through the continuous use of pasteurized and sterilized milk. Relying on our CR results, milk consumers in 33(68.75%) studies were exposed to moderate to high level of carcinogenic risk through the intake of Cd, Pb, As, and Hg.

The presence of heavy metals contaminants in the pasteurized and sterilized milk show that they are currently used illegally in both animal husbandry and agriculture. likewise, pasteurization and sterilization processes are not efficient for the elimination or degradation of the heavy metals addressed. In this sense, strict food safety laws in their correct spirit and letter should be made mandatory at every stage of milk processing units and handling to regulate and eliminate the prevalence heavy metals under permissible limits. Further studies are required to develop safe milk processing and handling methods for the decontamination of heavy metals in milk and its products.

**Competing interests**

There is no conflict of interest to declare.

**Funding**

The authors did not receive support from any organization for the submitted work. No funding was received to assist with the preparation of this manuscript.

**Reference**

1.	Teymouri Yeganeh M, Teymoury Yeganeh L. Evaluation of knowledge, practice and attitude of food health and safety in food service centers of Kermanshah province during the outbreak of Covid disease 19. Journal of Research in Environmental Health. 2021;7(3):292-300.

2.	Abedi S, Mohseni S, Mohammadi A, Makder A, Izadi N, Abedini S. Students’ knowledge, attitude, and practice regarding healthy and safe food in Hormozgan University of Medical Sciences. Journal of Preventive Medicine. 2019;6(2):73-66.

3.	Fakhreddini M, Tabib K, Amidi S, Kobarfard F, Tabatabai SA. Simultaneous measurement of lead and cadmium in the milk distributed in Tehran’s schools by differentialpulse anodic stripping voltammetry. Int Pharm Acta. 2019;2:e3.

4.	Ahmed M, Khaleeq A, Huma R, Qadir MA, Shafiq MI, Israr A, et al. Optimization and validation procedure for elemental composition of fresh and pasteurized milk in Pakistan employing microwave digestion followed by ICP-OES: A contribution to risk assessment. Food Analytical Methods. 2016;9:2933-42.

5.	Sharifi S, Sohrabvandi S, Mofid V, Khanniri E, Khorshidian N, Esmaeili S, et al. Determination of Lead Level in Pasteurized Milk and Dairy Products Consumed In Tehran and Evaluation of Associated Health Risk. International Journal of Cancer Management. 2021;14(9).

6.	Leksir C, Boudalia S, Moujahed N, Chemmam M. Traditional dairy products in Algeria: case of Klila cheese. Journal of Ethnic Foods. 2019;6(1):1-14.

7.	Shar Z, Pirhot O, Shar H, Channa M. Assessing the risk of heavy metals contamination in milk from Pakistan. Int J Sci Res Arch. 2021;3(01):107-13.

8.	Feizi R, Hamidi F, Jaafarzadeh N, Ghahrchi M, Zafarzadeh A. Determination and health risk assessment of heavy metals (Pb, Cd, Cu and Zn) in different brands of pasteurized milk. International Journal of Environmental Analytical Chemistry. 2022;102(18):6892-903.

9.	Nemati MH. Heavy metals in blood and milk of grazing animals around the vicinity of Zanjan zinc industrial park. Iranian Journal of Health and Environment. 2021;14(3):461-72.

10.	Fatehi MH, Shayegan J, Zabihi M. A review of methods for removing heavy metal from aqueous media. Iranian journal of Ecohydrology. 2018;5(3):855-74.

11.	Pajohi-Alamoti MR, Mahmoudi R, Sari AA, Valizadeh S, Kiani R. Lead and cadmium contamination in raw milk and some of the dairy products of Hamadan province in 2013-2014. Journal of Health. 2017;8(1):27-34.

12.	M Abd Elgawad M. Contamination and Health Risks of Certain Heavy Metals and Trace Elements in Milk and Milk Products Consumed in Province of Monufia, Egypt. Annals of Agricultural Science, Moshtohor. 2020;58(3):673-82.

13.	Eftekhari M, Shahrami E, Hadi Tavatori M, Atlasbaf M. Assessment of milk contamination by some heavy metals (lead, cadmium, chromium, nickel and mercury) of dairy cattle herd in Qazvin province and its effects on human health. Veterinary Researches & Biological Products. 2022;35(1):131-7.

14.	Shahbazi Y, Ahmadi F, Fakhari F. Validation of sequential cathodic-anodic stripping voltammetric determination of ultratrace level of copper, lead, cadmium, zinc, and selenium in milk and dairy products of Iran. Analytical and Bioanalytical Electrochemistry. 2016;8(8):948-61.

15.	Elbagermi MA, Haleem AB, Elsherif KM. Evaluation of essential and heavy metal levels in pasteurized and long-life cow milk. Int J Adv Chem. 2020;8(1).

16.	Sant'Ana MAR, de Carvalho TC, da Silva IF. Concentration of heavy metals in UHT dairy milk available in the markets of São Luís, Brazil, and potential health risk to children. Food Chemistry. 2021;346:128961.

17.	Shahbazi Y, Ahmadi F, Fakhari F. Voltammetric determination of Pb, Cd, Zn, Cu and Se in milk and dairy products collected from Iran: An emphasis on permissible limits and risk assessment of exposure to heavy metals. Food chemistry. 2016;192:1060-7.

18.	Sadeghi N, Behzad M, Homay Razavi S, Jannat B, Oveisi MR, Hajimahmoodi M. Measurement of zinc, copper, lead, and cadmium in the variety of packaging milk and raw milk in Tehran markets by anodic striping voltammetry. Journal of Chemical Health Risks. 2020;10(3):175-83.

19.	Birghila S, Dobrinas S, Stanciu G, Soceanu A. DETERMINATION OF MAJOR AND MINOR ELEMENTS IN MILK THROUGH ICP-AES. Environmental Engineering & Management Journal (EEMJ). 2008;7(6).

20.	Calahorrano-Moreno MB, Ordoñez-Bailon JJ, Baquerizo-Crespo RJ, Dueñas-Rivadeneira AA, Montenegro MCB, Rodríguez-Díaz JM. Contaminants in the cow's milk we consume? Pasteurization and other technologies in the elimination of contaminants. F1000Research. 2022;11.

21.	Sebastianski M, Bridger NA, Featherstone RM, Robinson JL. Disease outbreaks linked to pasteurized and unpasteurized dairy products in Canada and the United States: a systematic review. Canadian Journal of Public Health. 2022;113(4):569-78.

22.	Mancebo AM, Dorella MR, de Abreu SS, Carrilho SM, Ferreira GF, Itida RM, et al. Pasteurized milk quality in Brazil: a cross-sectional study over five years. The Journal of dairy research. 2023:1-4.

23.	Fleming PS, Koletsi D, Pandis N. Blinded by PRISMA: are systematic reviewers focusing on PRISMA and ignoring other guidelines? PLoS One. 2014;9(5):e96407.

24.	Organization WH. WHO human health risk assessment toolkit: chemical hazards. 2021.

25.	Noaman V, Rasti M, Heidari M, Ranjbari A, Allameh K. Evaluation of a group of organochlorine and organophosphorus pesticides residue and Lead content of row and pasteurized cow milk collected from Isfahan city, Iran. Veterinary Research & Biological Products. 2021;34(4):212-21.

26.	Harlia E, Rahmah K, Suryanto D, editors. Food safety of milk and dairy product of dairy cattle from heavy metal contamination. IOP conference series: earth and environmental science; 2018: IOP Publishing.

27.	Totan FE, Filazi A. Determination of some element levels in various kinds of cow’s milk processed in different ways. Environmental monitoring and assessment. 2020;192:1-10.

28.	Abdeljalil NA, Benkhayal AA, Ahmida M, El Sharaa IA, Ahmida NH. Determination of lead and cadmium in different types of milk samples collected from different markets in Benghazi-Libya. 2021.

29.	Rezaei M, Dastjerdi HA, Jafari H, Farahi A, Shahabi A, Javdani H, et al. Assessment of dairy products consumed on the Arakmarket as determined by heavy metal residues. Health. 2014;2014.

30.	Qin L-Q, Wang X-P, Li W, Tong X, Tong W-J. The minerals and heavy metals in cow's milk from China and Japan. Journal of health science. 2009;55(2):300-5.

31.	Ştefănescu L, Stezar CI, Groza IŞ. The influence of environmental contamination on heavy metals and organochlorine compounds levels in milk. Environmental Engineering and Management Journal. 2011;10(1):37-42.

32.	Algburi J, Jarrah N, Mohammed K, Jaafar A, Alshabani M, Almayahi B. Evaluation the Health Impact of Some Heavy Metals in Milk from Markets. Prensa méd argent. 2019:849-51.

33.	Abdelfatah EN, Mansour MA, Ahmed NI, El-Ganzory HH. Heavy metal residues and health risk assessment in raw milk and dairy products with a trail for removal of copper residues. Benha veterinary medical journal. 2019;36(1):51-64.

34.	Beikzadeh S, Ebrahimi B, Mohammadi R, Beikzadeh M, Asghari-Jafarabadi M, Foroumandi E. Heavy metal contamination of milk and milk products consumed in Tabriz. Current nutrition & food science. 2019;15(5):484-92.

35.	Akhtar S, Ismail T, Riaz M, Shahbaz M, Amin K. Minerals and heavy metals in raw and ultra heat treated commercial milks in Pakistan. International Journal of Food and Allied Sciences. 2015;1(1):18-24.

36.	Madani-Tonekaboni M, Rafiei Nazari R, Mirzamohammadi S, Abdolshahi A, Abbasi-bastami N, Arabameri M. Monitoring and risk assessment of lead and cadmium in milks from east of Iran using Monte Carlo simulation method. nutrition and food sciences research. 2019;6(2):29-36.

37.	Yu M, Liu Y, Achal V, Fu Q-L, Li L. Health Risk Assessment of Al and Heavy Metals in Milk Products for Different Age Groups in China. Polish Journal of Environmental Studies. 2015;24(6).

38.	Issa SY, Genena DM, Al-Mazroua MK, Abdel-Rahman S, Fawzi MM. Determination of some metals in the commonly consumed dairy products randomly collected from the market in Alexandria-Egypt, with an emphasis on toxicity, permissible limits and risk assessment. Int j pharmacol toxicol. 2016;4(2):133-7.

39.	Santos C, Oliveira A, Martins D, Oliveira J, Pedro F, Villa R. Determination of the concentrations of essential and toxic metals in UHT milk produced in Mato Grosso State, Brazil. International Food Research Journal. 2015;22(3).

40.	Suturović Z, Kravić S, Milanović S, Đurović A, Brezo T. Determination of heavy metals in milk and fermented milk products by potentiometric stripping analysis with constant inverse current in the analytical step. Food chemistry. 2014;155:120-5.

41.	Ghezelbash M, Darbani S, Rosta S, Majd A, Soltanolkotabi M, Azar A. Application of LIBS for identification and determination of element concentrations in milk. Iranian Journal of Nutrition Sciences & Food Technology. 2015;10(1):89-96.

42.	Ping J, Wang Y, Wu J, Ying Y. Development of an electrochemically reduced graphene oxide modified disposable bismuth film electrode and its application for stripping analysis of heavy metals in milk. Food Chemistry. 2014;151:65-71.

43.	Alibeigi A-N, Malakootian M, Mirzahoseini SA. Determine the amount of lead, cadmium, copper, zinc and calcium antagonists of milk and cheese which is produced in Kerman and Sirjan pasteurized milk factory. Journal of Environmental Science and Technology. 2016;18(3):13-23.

44.	Anetta L, Peter M, Agnieszka G, Jozef G. Concentration of selected elements in raw and ultra heat treated cow milk. The Journal of Microbiology, Biotechnology and Food Sciences. 2012;2(2):795.

45.	Khan N, Jeong IS, Hwang IM, Kim JS, Choi SH, Nho EY, et al. Analysis of minor and trace elements in milk and yogurts by inductively coupled plasma-mass spectrometry (ICP-MS). Food chemistry. 2014;147:220-4.

46.	Abd-El Aal SFA. Assessment of toxic heavy metals in some dairy products and the effect of storage on its distribution. Journal of American Science. 2012;8(8):655-70.

47.	De Castro CS, Arruda AF, Da Cunha LR, SouzaDe JR, Braga JW, Dórea JG. Toxic metals (Pb and Cd) and their respective antagonists (Ca and Zn) in infant formulas and milk marketed in Brasilia, Brazil. International Journal of Environmental Research and Public Health. 2010;7(11):4062-77.

48.	Lutfullah G, Khan AA, Amjad AY, Perveen S. Comparative study of heavy metals in dried and fluid milk in Peshawar by atomic absorption spectrophotometry. The scientific world journal. 2014;2014.

49.	AA AA, MA AD. Chemical composition, metals content and pesticide residues in raw, pasteurized and UHT milk and their dietary intake. 2008.

50.	Pohl P, Prusisz B. Determination of Ca, Mg, Fe and Zn partitioning in UHT cow milks by two-column ion exchange and flame atomic absorption spectrometry detection. Talanta. 2007;71(2):715-21.

51.	Kazi TG, Jalbani N, Baig JA, Kandhro GA, Afridi HI, Arain MB, et al. Assessment of toxic metals in raw and processed milk samples using electrothermal atomic absorption spectrophotometer. Food and Chemical Toxicology. 2009;47(9):2163-9.

52.	Tokuşoǧlu Ö, Aycan Ş, Akalin S, Koçak S, Ersoy N. Simultaneous differential pulse polarographic determination of cadmium, lead, and copper in milk and dairy products. Journal of agricultural and food chemistry. 2004;52(7):1795-9.

53.	Tripathi R, Raghunath R, Sastry V, Krishnamoorthy T. Daily intake of heavy metals by infants through milk and milk products. Science of the total environment. 1999;227(2-3):229-35.

54.	Zurera-Cosano G, Moreno-Rojas R, Amaro-Lopez M. Effect of processing on contents and relationships of mineral elements of milk. Food Chemistry. 1994;51(1):75-8.

55.	Sobhanardakani S. Human health risk assessment of Cd, Cu, Pb and Zn through consumption of raw and pasteurized cow’s milk. Iranian journal of public health. 2018;47(8):1172.

56.	Perveen F, Mumtaz M, Usmani T. Estimation of metal contents in different varieties of milk available in Karachi city. JOURNAL-CHEMICAL SOCIETY OF PAKISTAN. 2005;27(6):611.

57.	Soares VA, Kus MM, Peixoto ALC, Carrocci JS, Salazar RF, Izário Filho HJ. Determination of nutritional and toxic elements in pasteurized bovine milk from Vale do Paraiba region (Brazil). Food Control. 2010;21(1):45-9.

58.	Martino FAR, Sánchez MaLF, Sanz-Medel A. The potential of double focusing-ICP-MS for studying elemental distribution patterns in whole milk, skimmed milk and milk whey of different milks. Analytica chimica acta. 2001;442(2):191-200.

59.	GARCIÁ EM, LORENZO ML, CABRERA C, LÓPEZ MC, SÁNCHEZ J. Trace element determination in different milk slurries. Journal of dairy research. 1999;66(4):569-78.

60.	Rodrı, ́guez ER, ́guez, Alaejos MS, Romero CD, ́az. Mineral concentrations in cow's milk from the Canary Island. Journal of food composition and analysis. 2001;14(4):419-30.

61.	Fu Z, Xi S. The effects of heavy metals on human metabolism. Toxicology mechanisms and methods. 2020;30(3):167-76.

62.	Chenouf A, Chenouf N, Bouzidi A, Gaucheron F, Mati A. Heavy metals in small ruminant’s milk from Algerian area steppe. International Food Research Journal. 2016;23(3):1012-6.

63.	Tunegová M, Toman R, Tančin V. Heavy metals-environmental contaminants and their occurrence in different types of milk. Slovak Journal of Animal Science. 2016;49(3):122-31.

64.	Mihretu T. Determination of the levels of selected trace heavy metals and fat content in commercially available milk brands in Addis Ababa city. Ethiopia; 2018.

65.	Jaishankar M, Tseten T, Anbalagan N, Mathew BB, Beeregowda KN. Toxicity, mechanism and health effects of some heavy metals. Interdisciplinary toxicology. 2014;7(2):60.

66.	Järup L. Hazards of heavy metal contamination% J British Medical Bulletin. 68. 2003.

67.	European Commission E. Commission Regulation (EC) No 1881/2006 of 19 December 2006 setting maximum levels for certain contaminants in foodstuffs. Off J Eur Union. 2006;364:5-24.

68.	FAO W. Codex Alimentarius General standard for contaminants and toxins in food and feed CXS 193-1995. Adopted in. 1995.

69.	Joint F, Organization WH, Additives WECoF. Evaluation of certain food additives and contaminants: seventy-third [73rd] report of the Joint FAO/WHO Expert Committee on Food Additives: World Health Organization; 2011.

70.	Boudebbouz A, Boudalia S, Bousbia A, Habila S, Boussadia MI, Gueroui Y. Heavy metals levels in raw cow milk and health risk assessment across the globe: A systematic review. Science of the total Environment. 2021;751:141830.

71.	Briffa J, Sinagra E, Blundell R. Heavy metal pollution in the environment and their toxicological effects on humans. Heliyon. 2020;6(9).

72.	Standard I. International Dairy Federation Bulletin, Chemical residues in milk and milk products. IDF Document. 1979;133.

73.	Organization WH. Evaluation of certain food additives and contaminants: sixty-first report of the joint FAO/WHO expert committee on food additives. Evaluation of certain food additives and contaminants: sixty-first report of the joint FAO/WHO expert committee on food additives2004. p. 176-.

74.	Das A, Joardar M, Chowdhury NR, De A, Mridha D, Roychowdhury T. Arsenic toxicity in livestock growing in arsenic endemic and control sites of West Bengal: risk for human and environment. Environmental Geochemistry and Health. 2021;43:3005-25.

75.	Nath A, Samanta S, Banerjee S, Danda AA, Hazra S. Threat of arsenic contamination, salinity and water pollution in agricultural practices of Sundarban Delta, India, and mitigation strategies. SN Applied Sciences. 2021;3:1-15.

76.	Council NR. Critical aspects of EPA's IRIS assessment of inorganic arsenic: interim report: National Academies Press; 2013.

77.	Rahaman MS, Rahman MM, Mise N, Sikder MT, Ichihara G, Uddin MK, et al. Environmental arsenic exposure and its contribution to human diseases, toxicity mechanism and management. Environmental Pollution. 2021;289:117940.

78.	Palma-Lara I, Martínez-Castillo M, Quintana-Pérez J, Arellano-Mendoza M, Tamay-Cach F, Valenzuela-Limón O, et al. Arsenic exposure: A public health problem leading to several cancers. Regulatory Toxicology and Pharmacology. 2020;110:104539.

79.	Balali-Mood M, Naseri K, Tahergorabi Z, Khazdair MR, Sadeghi M. Toxic Mechanisms of Five Heavy Metals: Mercury, Lead, Chromium, Cadmium, and Arsenic. Frontiers in pharmacology. 2021;12:643972.

80.	Joint F, Organization WH, Additives WECoF. Evaluation of certain contaminants in food: seventy-second [72nd] report of the Joint FAO/WHO Expert Committee on Food Additives: World Health Organization; 2011.

81.	Rose M, Baxter M, Brereton N, Baskaran C. Dietary exposure to metals and other elements in the 2006 UK Total Diet Study and some trends over the last 30 years. Food Additives and Contaminants. 2010;27(10):1380-404.

82.	Aliasgharpour M, Rahnamaye Farzami M. Trace elements in human nutrition: A review. International journal of medical investigation. 2013;2(3):0-.

83.	COT. COT Statement on the 2006 UK Total Diet Study of Metals and Other Elements. HMSO London (UK); 2008.

84.	Aggarwal A, Verma T, Ghosh S. Heavy Metal Residues in Milk and Milk Products and Their Detection Method. Trends and Innovations in Food Science: IntechOpen; 2022.

85.	Joint F, Additives WECoF, Organization WH. Safety evaluation of certain contaminants in food: prepared by the Seventy-second meeting of the Joint FAO/WHO Expert Committee on Food Additives (JECFA): World Health Organization; 2011.

86.	Ukhurebor KE, Aigbe UO, Onyancha RB, Nwankwo W, Osibote OA, Paumo HK, et al. Effect of hexavalent chromium on the environment and removal techniques: a review. Journal of Environmental Management. 2021;280:111809.

87.	Lambiase S, Ariano A, Serpe FP, Scivicco M, Velotto S, Esposito M, et al. Polycyclic aromatic hydrocarbons (PAHs), arsenic, chromium and lead in warty crab (Eriphia verrucosa): occurrence and risk assessment. Environmental Science and Pollution Research. 2021;28:35305-15.

88.	den Braver-Sewradj SP, van Benthem J, Staal YC, Ezendam J, Piersma AH, Hessel EV. Occupational exposure to hexavalent chromium. Part II. Hazard assessment of carcinogenic effects. Regulatory Toxicology and Pharmacology. 2021;126:105045.

89.	Monga A, Fulke AB, Dasgupta D. Recent developments in essentiality of trivalent chromium and toxicity of hexavalent chromium: implications on human health and remediation strategies. Journal of Hazardous Materials Advances. 2022;7:100113.

90.	Pokhrel GR, Pokhre G. The Effect of Chromium on Human-Health: A Review. BMC Journal of Scientific Research. 2022;5(1):27-35.

91.	Kouokam JC, Meaza I, Wise JP. Inflammatory effects of hexavalent chromium in the lung: A comprehensive review. Toxicology and applied pharmacology. 2022:116265.

92.	EPA U. Integrated Risk Information System (IRIS): Chromium (VI)(CASRN 18540-29-9). US EPA. Support of Summary Information on the Integrated Risk Information …; 1998.

93.	Saúde BMd. Decreto n° 55.871 de 26 de março de 1965. Modifica o Decreto nº 50.040, de 24 de janeiro de 1961, referente a normas reguladoras do emprego de aditivos para alimentos, alterado pelo Decreto nº 691, de 13 de março de 1962. Diário Oficial da União; 1965.

94.	Silva CS, Moutinho C, Ferreira da Vinha A, Matos C. Trace minerals in human health: Iron, zinc, copper, manganese and fluorine. International Journal of Science and Research Methodology. 2019;13(3):57-80.

95.	William NG, Godfrey M, Savino B. Assessment of heavy metals in milk from selected dairy farms and shops in Wakiso District, Uganda. Suza Journal of Naturaal and Social Science. 2011;1(1):36-52.

96.	Organization WH. Trace elements in human nutrition and health: World Health Organization; 1996.

97.	Aras NK, Ataman OY. Trace element analysis of food and diet: Royal Society of Chemistry; 2007.

98.	RAR E. European Union Risk Assessment Report: Nickel and nickel compounds. Office for Official Publications of the European Communities. 2008.

99.	Umar M, Sultan A, Jahangir N, Saeed Z. Cobalt toxicity. Metal Toxicology Handbook: CRC Press; 2020. p. 287-301.

100.	Banerjee P, Bhattacharya P. Investigating cobalt in soil-plant-animal-human system: dynamics, impact and management. Journal of Soil Science and Plant Nutrition. 2021;21(3):2339-54.

101.	Dieu SG, Loubou ML, Joel GSC, Félix BAJ. Assessment of trace elements pollution and their potential health risks in the cobalt–nickel bearing areas of Lomié, East Cameroon. Environmental Monitoring and Assessment. 2022;194(2).

102.	Vaicelyte A, Janssen C, Le Borgne M, Grosgogeat B. Cobalt–Chromium dental alloys: Metal exposures, toxicological risks, CMR classification, and EU regulatory framework. Crystals. 2020;10(12):1151.

103.	Dolomatov SI, Sataeva T, Żukow W. Modern aspects of regulatory, pathophysiological and toxic effects of cobalt ions during oral intake in the human body. 2019.

104.	Meshref AM, Moselhy WA, Hassan NE-HY. Heavy metals and trace elements levels in milk and milk products. Journal of food measurement and characterization. 2014;8:381-8.

105.	Aguilar F, Autrup H, Barlow S, Castle L, Crebelli R, Dekant W, et al. Safety of aluminium from dietary intake scientific opinion of the panel on food additives, flavourings, processing aids and food contact materials (AFC). EFSA J. 2008;754:1-34.

106.	EFSA. Tolerable Upper Intake levels of vitamins and minerals by the Scientific Panel on Dietetic products, nutrition and allergies (NDA) and Scientific Committee on Food (SCF). 2006.

107.	‐ G. National Food Safety Standard Maximum Levels of Contaminants in Foods. Ministry of Health of the People’s Republic of China Beijing, China; 2012.

108.	Organization WH. Joint FAO/WHO Food Standards Programme Codex Committee on Contaminants in Food. The Hague, The Netherlands. 2011.

109.	Zaynab M, Al-Yahyai R, Ameen A, Sharif Y, Ali L, Fatima M, et al. Health and environmental effects of heavy metals. Journal of King Saud University-Science. 2022;34(1):101653.

110.	Asayehegn AT, Hymete A, Hagos S, Ashenef A. Determination of the Level of Heavy Metals in Cow’s Milk Collected from Butajirra and Meskan Districts, South Central Ethiopia. 2021.

111.	Ziarati P, Shirkhan F, Mostafidi M, Zahedi MT. An overview of the heavy metal contamination in milk and dairy products. Acta scientific pharmaceutical sciences. 2018;2(7):1-14.

112.	Al-Masri M, Al-Kharfan K, Al-Shamali K. Speciation of Pb, Cu and Zn determined by sequential extraction for identification of air pollution sources in Syria. Atmospheric Environment. 2006;40(4):753-61.

113.	CONI E, BOCCA B, CAROLI S. Minor and trace element content of two typical Italian sheep dairy products. Journal of dairy research. 1999;66(4):589-98.

114.	Mahmoudi R, Kazeminia M, Kaboudari A, Pir-Mahalleh S, Pakbin B. A review of the importance, detection and controlling of heavy metal in milk and dairy products. Malaysian Journal of Science. 2017;36(1):1-16.

115.	Organization WH. Evaluation of certain food additives and contaminants: sixty-seventh report of the Joint FAO/WHO Expert Committee on Food Additives. Evaluation of certain food additives and contaminants: sixty-seventh report of the Joint FAO/WHO Expert Committee on Food Additives2007. p. 94-.
